# Emerging virulent clones of community-onset *Acinetobacter baumannii* in Taiwan

**DOI:** 10.1186/s41182-025-00850-1

**Published:** 2025-12-16

**Authors:** Tu Quyen Tran Lam, Yu-Chia Hsieh, Thi Tuyet-Anh Nguyen, I.-Hsin Sung, Shiao-Wen Li, Yi-Jiun Pan

**Affiliations:** 1 Department of Biological Science and Technology, College of Life Science, China Medical University, Taichung, Taiwan; 2 Department of Microbiology and Immunology, School of Medicine, College of Medicine, China Medical University, Taichung, Taiwan; 3 Department of Pediatrics, Chang Gung Children’s Hospital, Chang Gung Memorial Hospital, Chang Gung University, College of Medicine, Taoyuan, Taiwan; 4 Department of Plant Medicine, National Chiayi University, Chiayi City, Taiwan; 5 Department of Life Sciences, National University of Kaohsiung, Kaohsiung, Taiwan

**Keywords:** Community-onset *Acinetobacter baumannii* (COAB), Capsular types (KLs), Multi-locus sequence typing (MLST), Virulent phenotypes, *Galleria mellonella* larvae model

## Abstract

**Background:**

*Acinetobacter baumannii* has emerged as a significant global pathogen, and community-acquired infections are concerning due to their severe clinical outcomes and high mortality. Despite this, the molecular epidemiology and phenotypic characteristics of community-acquired/community-onset *A. baumannii* (CAAB/COAB) strains remain poorly understood. This study analyzed the genotypes, virulence traits, and clinical manifestations of 32 COAB isolates collected in Taiwan between 2015 and 2017.

**Methods:**

Capsular types (KLs), sequence types (STs) from the Oxford and Pasteur schemes, and international clones (ICs) were identified among the 32 COAB isolates. In vitro virulence was assessed by evaluating biofilm formation, motility, resistance to desiccation and serum, and in vivo virulence was confirmed in a *Galleria mellonella* larvae model. Associations between KL/ST types and virulence phenotypes, as well as between KL/ST types and the clinical manifestations of patients, were also analyzed.

**Results:**

The results showed that among the tested COAB isolates, KL49 was the predominant capsular type, representing 18.8% (*n* = 6) of samples, and ST10^Pas^/IC8 (ST10^Pas^: ST10 under the Pasteur scheme, IC8: international clone 8) was the major clone (15.6%, *n* = 5). Interestingly, we found that KL49/ST10^Pas^, which is predominant in America and Australia but has never been reported for CAAB/COAB in Taiwan, had a hypervirulent phenotype with high serum resistance and high mortality in the *G. mellonella* larvae model. Furthermore, clinical records showed higher incidences of chronic obstructive pulmonary disease, pneumonia, elevated Pitt bacteremia scores, and 30-day mortality for patients with KL49/ST10^Pas^ infections than for patients with non-KL49/ST10^Pas^ infections.

**Conclusions:**

This is the first report identifying KL49/ST10^Pas^ as a major clone of COAB in Taiwan. Its high virulence was demonstrated, highlighting a potential public health threat. This study lays a foundation for understanding the molecular epidemiology of COAB in Taiwan and supports future research on virulence and disease control strategies.

**Supplementary Information:**

The online version contains supplementary material available at 10.1186/s41182-025-00850-1.

## Introduction

*Acinetobacter baumannii* is a notable opportunistic pathogen associated with a wide range of nosocomial and community-acquired infections, which lead to substantial morbidity and mortality [1–3]. Despite its lower antibiotic resistance rate than hospital-acquired *A. baumannii* (HAAB), community-acquired *A. baumannii* (CAAB)/community-onset *A. baumannii* (COAB) is increasingly recognized as a serious health concern, as it frequently causes rapidly progressive pneumonia with high mortality, particularly in individuals with certain risk factors, such as chronic alcohol use, smoking, and residing in tropical regions [4, 5]. Although the mechanisms underlying the aggressive nature of CAAB/COAB remain unclear, the distinct clinical features of CAAB/COAB infections, including high rates of bacteremia, adult respiratory distress syndrome (ARDS), disseminated intravascular coagulopathy (DIC), and mortality, raise questions about the roles of host susceptibility, bacterial virulence, or both in its aggressiveness [6–8].

Whether the community serves as a reservoir for endemic and epidemic hospital strains is under debate. Some studies have provided evidence of genetic relatedness between CAAB/COAB and HAAB [9, 10]. Meumann et al. compared whole-genome sequences (WGS) of local COAB and HAAB strains from northern Australia to global strains in the NCBI database and found that COAB and nosocomial strains shared genetic characteristics [10]; this study also reported that Pasteur sequence type (ST)10 was the dominant ST in both COAB and HAAB strains. In contrast, other studies reported differences between CAAB and HAAB strains. A study of 32 CAAB isolates from West China identified 11 clonal patterns, which differed from the predominantly clonal dissemination observed in hospitals [11]. Similarly, Zeana C. et al. showed that *A. baumannii* isolates from a community in the USA were characterized by high diversity, with mostly unrelated strains that were distinct from hospital isolates [12]. In an Australian study of a CAAB strain, a complete genome and phenome analysis identified the strain as ST type ST267 (Pasteur) and showed that the phylogenetic relatives of this strain did not belong to either the international clone (IC)1 or IC2 lineages, which are the predominant lineages of hospital isolates [13]. These studies were consistent with the notion that community-acquired isolates are epidemiologically distinct from nosocomial isolates.

In *A. baumannii*, the capsule is a crucial virulence factor that contributes to its resistance against complement-mediated killing, serves as a barrier to immune recognition, and enhances desiccation tolerance, motility, cell adherence, and biofilm production [14–17]. To date, over 200 capsular types (KL types) have been identified, and different properties of KL types have been noted [15, 18, 19]. In our previous study of HAAB isolates in Taiwan, we showed that patients infected with carbapenem-resistant *A. baumannii* (CRAB) strains of the four major KL types (KL2, KL10, KL22, and KL52) had significantly worse outcomes than those infected with CRAB strains of other KL types [20]. Another study of 220 HAAB isolates in China reported KL2 as the most common KL type, which had significantly higher resistance to all antimicrobials but lower biofilm formation than non-KL2 isolates [16]. A study from Thailand revealed that almost 90% of global clonal 2 (GC2) HAAB isolates were types KL6, KL10, and KL47, and these types were resistant to human serum complement [21]. Despite the importance of certain capsular types of *A. baumannii*, most research has predominantly focused on HAAB isolates, leaving significant gaps in understanding the contribution of KL types to virulence in CAAB/COAB strains.

Although studies of CAAB/COAB infections from Réunion Island [22], China [11, 23], Malaysia [24], Australia [4, 10], and North America [7] have reported severe symptoms and poor clinical outcomes in infected patients, the molecular epidemiology and virulence characteristics of these strains are still underexplored. In Taiwan, research also has predominantly focused on HAAB isolates. Only a few studies of CAAB/COAB have been reported, which analyzed isolates collected before 2010 and lacked deeper investigations, such as analyses of virulence and identification of ST/KL types [25–27]. A 2002 study of 14 CAAB isolates collected in 1994–1999 and reported that all isolates were genetically distinct by PFGE analysis, indicating that there were no major clones spreading within the community in Taiwan [26]. A retrospective matched case–control study conducted in Taiwan of CAAB and HAAB isolates collected over a 12-year period (1999–2010) compared their clinical, epidemiologic, and microbiological characteristics and found that bacteremia caused by CAAB was associated with severe outcomes, warranting further investigation into the virulence factors of CAAB [27]. However, the number of more recent studies on CAAB/COAB is limited, and the reported information on isolates collected after 2010 lacks sufficient detail. Therefore, in this study, we investigated the molecular epidemiology, genotype-associated clinical outcomes, and virulence traits of COAB isolates collected in Taiwan from 2015 to 2017, to provide important information on these understudied strains.

## Methods

### Study population

This study included 32 COAB isolates collected from patients at the Lin Kou branch of Chang Gung Memorial Hospital (CGMH), a 3,700-bed medical center in northern Taiwan, and the Kaohsiung branch of CGMH, a 2,700-bed medical center in southern Taiwan, between 2015 and 2017. Community-onset bacteremia was defined as the presence of at least one positive blood culture from a patient exhibiting signs and symptoms of infection, collected within 48 h after admission, regardless of prior healthcare exposure. For patients with multiple episodes of bacteremia, only the isolate from the first episode was included. This protocol was approved by the institutional review board of CGMH (Approval number: 201801433B0). *A. baumannii* was identified using matrix-assisted laser desorption-time of flight mass spectrometry (MALDI-TOF-MS) [20, 28] and *bla*_OXA-51-like_ PCR [29, 30]. Antimicrobial susceptibility testing was determined using the disk diffusion method according to Clinical and Laboratory Standards Institute (CLSI) criteria [31]. Minimal inhibitory concentrations (MICs) for critical drugs, including carbapenem (imipenem and meropenem) and colistin, were determined using the broth microdilution method following CLSI guidelines. Resistance was defined as an MIC > 4 mg/L for carbapenem and ≥ 4 mg/L for colistin.

We selected 24 HAAB isolates for comparison with the COAB strains. These included 21 isolates that were randomly selected to represent the four predominant KL types—KL2 (*n* = 7), KL10 (*n* = 5), KL22 (*n* = 4), and KL52 (*n* = 5)—as well as three isolates from other KL types (KL-others). All isolates were obtained from a previous study conducted in Taiwan [20].

### Data collection

Patient characteristics, including demographic information and clinical outcomes, were retrospectively extracted from medical records. This study was conducted following the ethical principles of the Declaration of Helsinki and was approved by the Institutional Review Board of Chang Gung Memorial Hospital (Approval number: 201801433B0).

### Genotyping

#### Capsular typing using multiplex *wzy* and *wzc/wzy* polymerase chain reaction (PCR)

We screened the 32 COAB isolates for some common KL types using multiplex *wzy* PCR as previously described [32]. For those with undetectable KL types, *wzc* typing was performed [20]. When *wzc* sequences were obtained, they were compared to reference strains using NCBI BLAST to identify potential KL types. Subsequently, KL types were confirmed with type-specific *wzy* primers.

#### Multi-locus sequence typing

Multi-locus sequence typing (MLST) of 32 COAB strains was conducted using both the Oxford and Pasteur schemes [33, 34]. The primers and PCR conditions used were described in a previous study [35]. PCR amplicons were sequenced and analyzed using the public databases for molecular typing and microbial genome diversity (PubMLST) to determine the sequence types (STs) (https://pubmlst.org/). The ST clustering of various KL types was visualized using the web-based tool PHYLOVIZ (https://online2.phyloviz.net/index).

The evolutionary relationship between ST types and the major clonal complexes of COAB strains was analyzed using the goeBURST (global optimal Based Upon Related Sequence Types) algorithm (http://www.phyloviz.net/goeburst/), with link colors indicating levels of genetic similarity and the application of tiebreak rules [36]: black links represent unambiguous Single Locus Variants (SLVs) requiring no tiebreak rules; blue links denote SLVs resolved using Tiebreak Rule 1, prioritizing STs with the highest SLV count; gray links indicate more distant relationships: darker gray for double locus variants (DLVs) and lighter gray for triple locus variants (TLVs); and yellow links resolve ties remaining after Rule 1 using Rule 4 (ST frequency in the dataset) or Rule 5 (smallest ST identifier).

#### Single-locus ***bla***_OXA-51-like_ sequence-based typing (SBT)

MLST, a genotyping method based on housekeeping gene variants, has been widely applied. The *bla*_OXA-51-like_ gene family, which is intrinsic to *A. baumannii*, is a reliable genetic marker for identifying ICs [37]. Thus, combining MLST with sequence variations of *bla*_OXA-51-like_ is thought to enhance discriminatory power, allowing for more precise differentiation between closely related clones. The primer pairs and PCR conditions were as described previously [29]. The *bla*_OXA-51-like_ variants were verified using the beta-lactamase database (BLDB; http://www.bldb.eu/) [37].

### Biofilm formation assay

Biofilm formation was assessed as previously described, with slight modifications [38]. Overnight bacterial cultures were diluted 1:1000 in Luria–Bertani (LB) broth, and 200-μL aliquots of the diluted cultures were transferred to 96-well polystyrene plates and then incubated at 37 °C for 24 h without shaking. LB broth served as the negative control. After incubation, the wells were washed three times with 0.95% NaCl to remove non-adherent bacteria, and the adherent bacteria were stained with 200 μL of 0.5% crystal violet for 10 min. Excess stain was washed away with 0.95% NaCl, and then bound dye was solubilized with 180 μL of 33% glacial acetic acid. Biofilm formation was quantified by measuring the optical density (OD) at 570 nm. These experiments were conducted in triplicate.

### Desiccation assay

As previously described [15], overnight bacterial cultures were diluted 1:1000, and 25-μL aliquots (containing approximately 10^4^ CFU) were placed onto a 96-well plate at room temperature. After 15 days, the wells were rehydrated with 100 μL of sterile 1 × PBS for 30 min, followed by serial dilution and plating onto LB agar to enumerate surviving bacteria.

### Motility assays

Swarming and twitching motility assays were performed using the subsurface agar method as previously described, with some modifications [21, 39]. Swarming motility was assessed by inoculating freshly cultured bacteria onto LB agar (0.4%) containing 10% 2,3,5-triphenyltetrazolium chloride (TTC), while twitching motility was evaluated at the interface of a Petri dish with a 0.8% agar layer. The plates were incubated at 37 °C for 48 h. Swarming was considered positive if the zone was ≥ 10 mm. To assess twitching, the agar was removed after incubation, and the bacteria were stained with 0.1% crystal violet; a positive result was defined as a stained zone ≥ 5 mm. These experiments were conducted in triplicate.

### Serum bactericidal tests

Normal human serum (NHS) was pooled from healthy donors and stored at −80 °C until use. Susceptibility to normal human serum (NHS) was assessed as described previously, with slight modifications [21, 40]. A 25-µL aliquot of a bacterial cell suspension (containing 2.5 × 10^4^ CFU) was mixed with 75 µL of fresh NHS and incubated at 37 °C for 3 h without shaking. Serial dilutions were plated on LB agar to count surviving bacteria. These experiments were conducted in triplicate.

### In vivo* G. mellonella* model for assessing bacterial pathogenesis

The *G. mellonella* killing assay was conducted according to a previously described method, with some modifications [41]. Briefly, overnight bacterial cultures were centrifuged at 6,000× *g* for 6 min, and the bacterial pellet was resuspended in 1 mL of 1 × PBS to an OD at 600 nm of approximately 0.2–0.3 (≈2.5 × 10⁷ CFU/mL). *G. mellonella* larvae were injected with 20 μL of a bacterial suspension containing approximately 5 × 10^5^ CFU into the last left proleg. The control groups consisted of (1) larvae injected with 1 × PBS without bacteria and (2) non-manipulated larvae to evaluate background mortality. The larvae were incubated in petri dishes at 37 °C for 8 days and observed for survival every 24 h. The experiments were conducted in triplicate, each with eight larvae per group. As a control for virulence assessment, the well-characterized reference strain *A. baumannii* ATCC19606, which exhibits low virulence in both *G. mellonella* and murine infection models [42–44], was included.

### Statistical analysis

All statistical analyses were conducted using GraphPad Prism version 8.0.1 (GraphPad Software, San Diego, CA, USA). Data from the in vitro virulence assays, including biofilm formation, desiccation, motility, and serum bactericidal tests, were analyzed with Welch’s t-test to assess statistical significance between two groups (e.g., KL49 vs. non-KL49, ST10^Pas^ vs. non-ST10^Pas^, and COAB vs. HAAB). The survival curve of *G. mellonella* larvae was constructed using the Kaplan–Meier method, with *p-*values calculated by the log-rank test. The demographic data and clinical manifestations of 32 patients infected with bacterial strains from two groups (KL49 vs. non-KL49; and ST10^Pas^ vs. non-ST10^Pas^) were compared by Welch’s t-test for continuous variables and Fisher’s exact test for categorical variables. Differences in antibacterial susceptibility between COAB and HAAB isolates were also assessed using Fisher’s exact test. Statistical significance was defined as a *p*-value < 0.05. The statistical power was calculated using G*Power 3.1.9.7 (https://www.psychologie.hhu.de/arbeitsgruppen/allgemeine-psychologie-und-arbeitspsychologie/gpower).

## Results

### Genetic features of COAB isolates

**Fig. 1:**
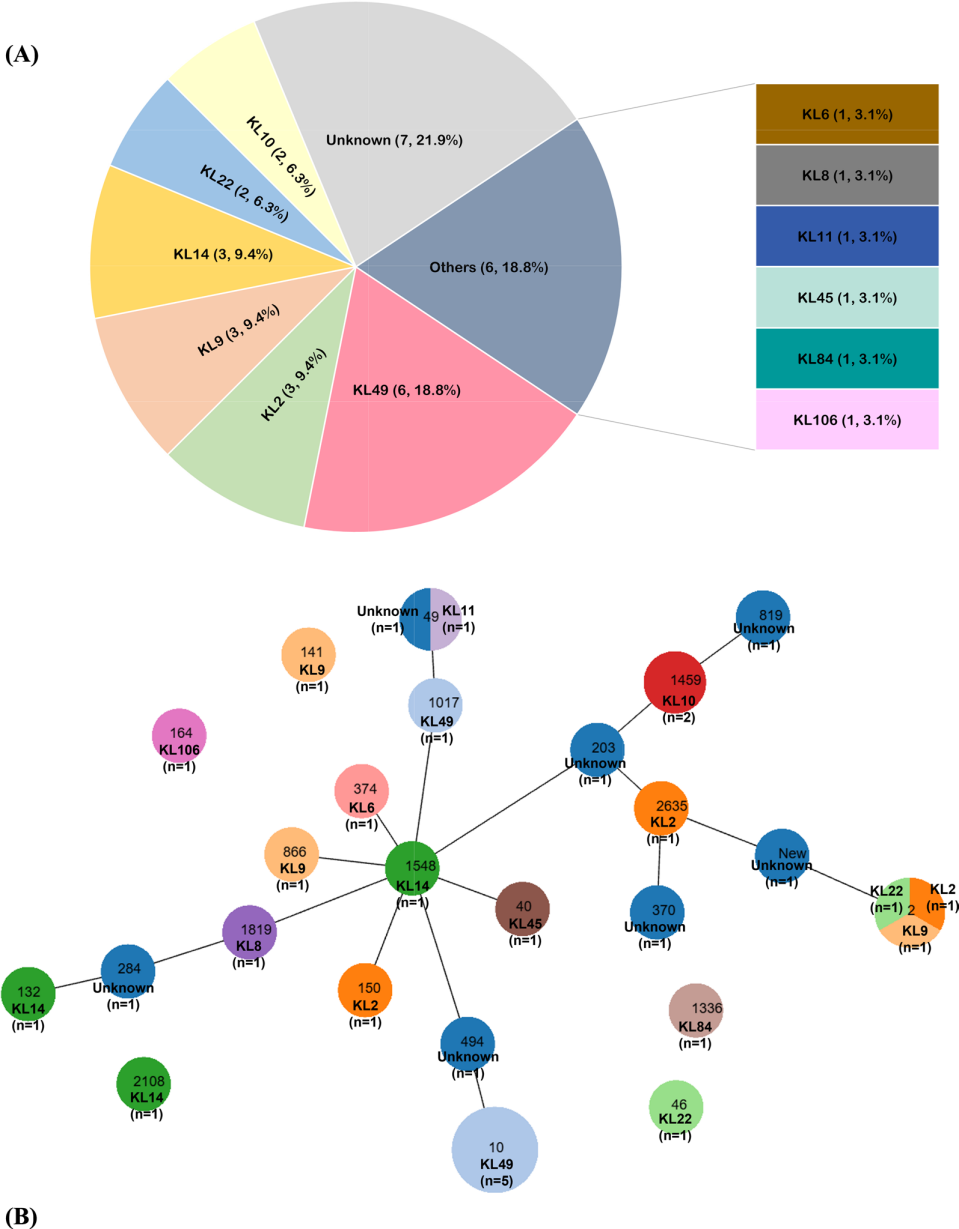
Genotyping of 32 community-onset *Acinetobacter baumannii* (COAB) isolates. **A** The prevalence of capsular (KL) types, **B** PHYLOViZ shows the distribution of KL types within the ST clusters of 32 COAB isolates with identified ST^Pas^ types. Each cake represents one ST^Pas^ type, and different KL types are shown in different colors. The node size varies linearly with the number of isolates of a given ST. The number of isolates is displayed in parentheses

A total of 32 COAB isolates were collected at two hospitals in Taiwan from 2015–2017. Identification of KL types using multiplex *wzy* and *wzc/wzy* PCR revealed KL49 as the most prevalent type (18.8%, *n* = 6), followed by KL9, KL14, and KL2 (each at 9.4%, *n* = 3), and KL10 and KL22 (each at 6.3%, *n* = 2). Six other types, each containing one strain, were grouped as “Other KL types” (18.8%; *n* = 6), and the remaining seven strains, which we were unable to identify using either multiplex *wzy* or *wzc* typing, were noted as “unknown types” (Fig. 1A).

The sequence types (ST) of the 32 strains were determined using two methods, the Pasteur and Oxford schemes. The former method, which better identifies clonal lineages, is used to compare evolutionarily distant clones but with lower discrimination, whereas the latter method is more effective for discriminating among strains at shorter evolutionary distances [33]. The allele profiles of the seven housekeeping genes used for both the Pasteur and Oxford schemes are detailed in Table S1.

The Pasteur scheme identified 23 STs among the identified KL types (Table 1), with numbers ranging from 1 to 5. The most common STs were ST10 (*n* = 5), which is the ST predominantly reported in America and Australia [10, 45], followed by ST2 (*n* = 3), ST49 (*n* = 2), and ST1459 (*n* = 2), while 19 other STs, and one newly identified in this study, were represented by a single isolate each. The clustering of these STs was visualized using goeBURST v.1.2.1 analysis. The goeBURST results showed that the 32 isolates in 18 STs could be classified into diverse groups, with ST132 as a group founder, and ST1548, ST203, and ST2635 as subgroup founders. Five identified STs were singletons without any association with other COAB isolates (Fig. S1A). We also grouped HAAB isolates from Taiwan in 71 ST^Pas^ types in the PubMLST database using goeBURST. This analysis identified ST2 as a group founder, with ST195 and ST719 as subgroup founders (Fig. S1B). ST134, ST377, ST388, ST725, and ST726 were also identified as subgroup founders, while eight STs were singletons. Notably, ST2, ST132, ST141, and ST374 were identified in both COAB and HAAB isolates in Taiwan, whereas ST10, the predominant type among COAB isolates, together with several other unique STs, was found exclusively in community-onset isolates. A detailed list of all STs for COAB and HAAB isolates is presented in Table S2.

**Table 1:** Distribution of ST types, *bla*_OXA-51_ variants, IC types for each KL type

KL type	STs^Oxford^	STs^Pasteur^	*bla*_OXA-51_ variants	IC type
KL49 (6)	ST-New1 (1)	ST10 (1)	OXA-68 (1)	IC8
	ST-New2 (2)	ST10 (2)	OXA-1067 (1)	IC8
			OXA-68 (1)	
	ST-New3 (1)	ST10 (1)	OXA-68 (1)	IC8
	ST-New4 (1)	ST10 (1)	OXA-68	IC8
	ST-New5 (1)	ST1017 (1)	OXA-855 (1)	^a^NoIC
KL2 (3)	ST208 (1)	ST2 (1)	OXA-508 (1)	IC2
	ST744 (1)	ST150 (1)	OXA-121 (1)	NoIC
	ST-New6 (1)	ST2635 (1)	OXA-1077 (1)	NoIC
KL9 (3)	ST218 (1)	ST2 (1)	OXA-66 (1)	IC2
	ST2152 (1)	ST141 (1)	OXA-91 (1)	NoIC
	ST1436 (1)	ST866 (1)	OXA-385 (1)	NoIC
KL14 (3)	ST-New7 (1)	ST132 (1)	OXA-120 (1)	NoIC
	ST1199 (1)	ST1548 (1)	OXA-259 (1)	NoIC
	ST2696 (1)	ST2108 (1)	OXA-65 (1)	NoIC
KL22 (2)	ST473 (1)	ST2 (1)	OXA-508 (1)	IC2
	ST-New8 (1)	ST46 (1)	OXA-104 (1)	NoIC
KL10 (2)	ST-New9 (1)	ST1459 (2)	OXA-98 (2)	NoIC
	ST-New10 (1)			
KL6 (1)	ST1416	ST374	OXA-259	NoIC
KL8 (1)	ST-New11	ST1819	OXA-769	NoIC
KL11 (1)	ST-New12	ST49	OXA-98	NoIC
KL45 (1)	ST-New13	ST40	OXA-685	NoIC
KL84 (1)	ST-New14	ST1336	OXA-555	NoIC
KL106 (1)	ST-New15	ST164	OXA-91	NoIC
Unknown (7)	ST-New16 (1)	ST49 (1)	OXA-1077 (2)	NoIC
	ST-New17 (1)	ST203 (1)		
	ST-New18 (1)	ST284 (1)	OXA-120 (1)	
	ST-New19 (1)	ST370 (1)	OXA-51 (1)	
	ST-New20 (1)	ST494 (1)	OXA-340 (1)	
	ST-New21 (1)	ST819 (1)	OXA-65 (1)	
	ST-New22 (1)	ST-New (1)	OXA-856 (1)	

The number of each type is shown in parentheses

^a^NoIC: refers to an IC type that does not belong to any of the currently defined International Clones (IC1–IC9)

When the Oxford scheme (which is more discriminative than Pasteur scheme) was used, the diversity of strains was further revealed, as each strain belonged to a different ST Oxford type (Table 1). Notably, 22 STs were newly identified and reported for the first time in this study, with 21 STs exhibiting novel combinations of previously known alleles, and one ST featuring a new allele of the *gpi* gene, resulting in a unique allelic profile (Table S1). The relationships between these STs were illustrated using goeBURST analysis, which revealed many scattered STs and four small groups (Fig. S1C).

The global spread of *A. baumannii* is driven by several high-risk international clones (ICs) [46]. Identifying ICs of *A. baumannii* is crucial for understanding its epidemiology and spread. Traditionally, isolates are assigned to specific ICs using WGS, which requires advanced computational expertise and may present challenges due to varying definitions across studies. To address these issues, a streamlined approach has been developed that combines ST^Pas^ types with an analysis of intrinsic *bla*_OXA-51-like_ variants [37]. Thus, we identified the *bla*_OXA-51-like_ variants, and all the OXA-51-like alleles present are listed in Table 1. Among the 32 COAB isolates, only eight could be classified into documented IC types, with five KL49 strains characterized as IC8 and three strains presenting different KL types belonging to IC2.

To investigate the association between KL and ST types, ST^Pas^ clustering of various KL types was visualized with the PHYLOViZ web-based tool (Fig. 1B). The results show that some strains with the same KL type were closely clustered, while some showed greater diversity. For example, the most prevalent KL type among the 32 COAB isolates, KL49, was strongly associated with ST10^Pas^, with five out of six being ST10^Pas^ strains, and the remaining strain being ST1017^Pas^. For the other KL types, i.e., KL9, KL14, KL2, and KL22, the strains belonging to the same type were scattered in different STs.

### Biofilm formation, desiccation, and motility of KL49–ST10^Pas^

**Fig. 2:**
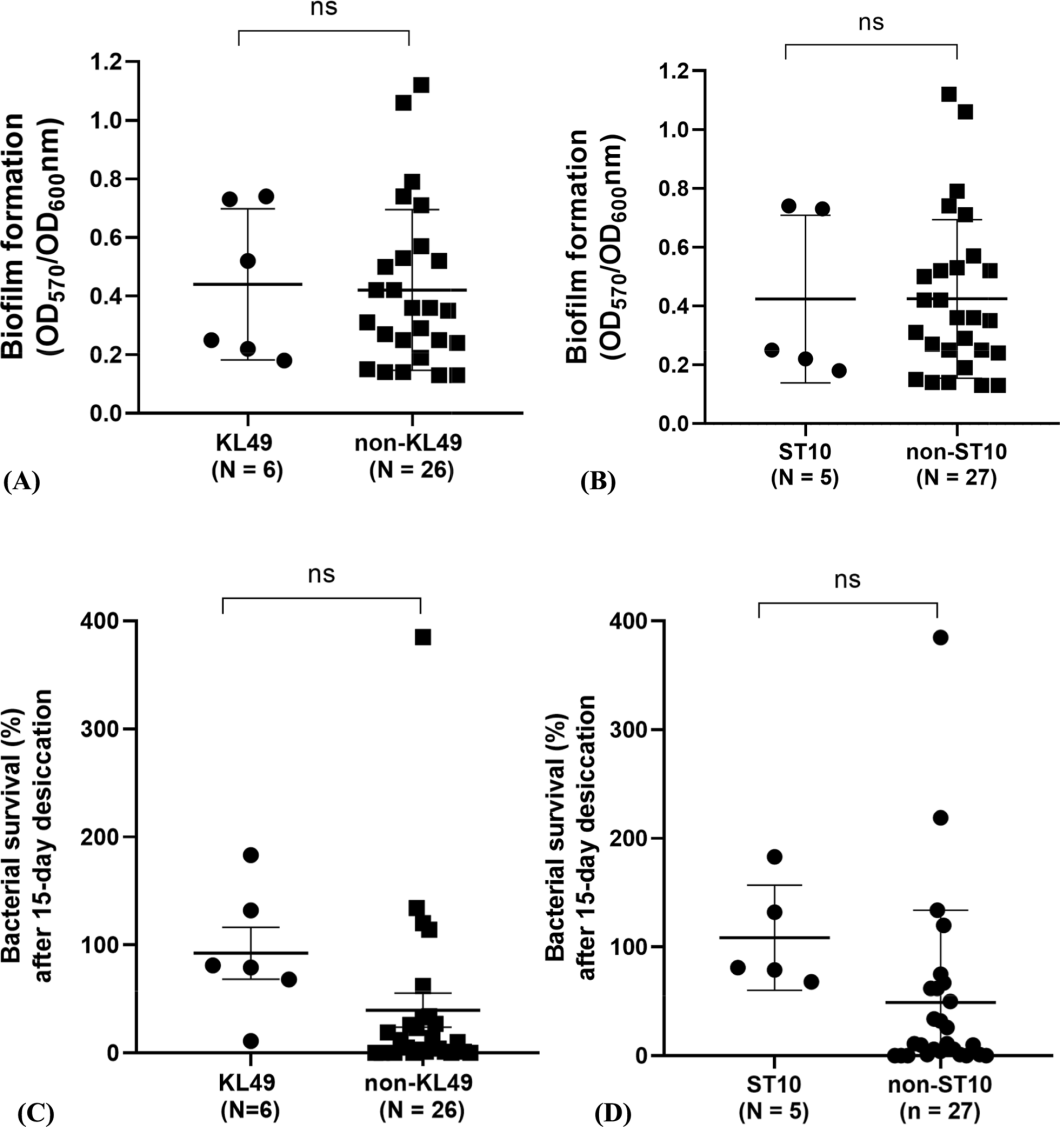
Biofilm formation and desiccation resistance of COAB isolates. Comparison of biofilm formation **A** between KL49 and non-KL49 isolates and **B** between ST10^Pas^ and non-ST10^Pas^ isolates**.** Comparison of resistance to dryness **C** between KL49 and non-KL49 isolates and **D** between ST10^Pas^ and non-ST10^Pas^ isolates. *p-*values were calculated using Welch's t-test (ns = not significant, *p* > 0.05)

To understand the significance of the major clone KL49/ST10^Pas^, its virulence characteristics were investigated. First, we analyzed the biofilm formation ability of the 32 COAB strains to investigate whether KL49 or ST10^Pas^ strains exhibit higher biofilm formation than the others. The results indicated that the COAB isolates exhibited a wide range of biofilm formation abilities, and there was no significant difference between KL49 and non-KL49 strains (Fig. 2A) or ST10^Pas^ and non-ST10^Pas^ strains (Fig. 2B). The distribution of biofilm formation abilities among the different KL and ST types is shown in Figure S2A and S2B, respectively. Additionally, a subset of 24 randomly selected HAAB strains from a previously documented collection in Taiwan, including four major KL types (KL2, KL10, KL22, and KL52) as well as several other KL types, was included for comparison with the COAB strains [20, 32]. Interestingly, the HAAB isolates exhibited higher biofilm production than the COAB strains (*p* = 0.0042, Fig. S2C).

Desiccation tolerance enables *A. baumannii* to thrive in hostile environments, and capsule formation has been reported as a contributor to this attribute [15]. Here, we investigated whether KL49 or ST10^Pas^ strains exhibited higher desiccation tolerance than the other strain types. After 15 days of desiccation, although there were no significant differences in viability between either KL49 and non-KL49 strains (Fig. 2C) or between ST10^Pas^ and non-ST10^Pas^ strains (Fig. 2D), the average viability of ST10^Pas^ strains was about 100%, whereas those of non-ST10^Pas^ groups was about 49% (Fig. S3A). The distributions of the desiccation tolerance among different KL and ST types are shown in Figures S3B and S3C, respectively. Notably, the desiccation ability of the COAB isolates was significantly higher than that of the HAAB isolates (*p* = 0.0015, Fig. S3D).

**Fig. 3:**
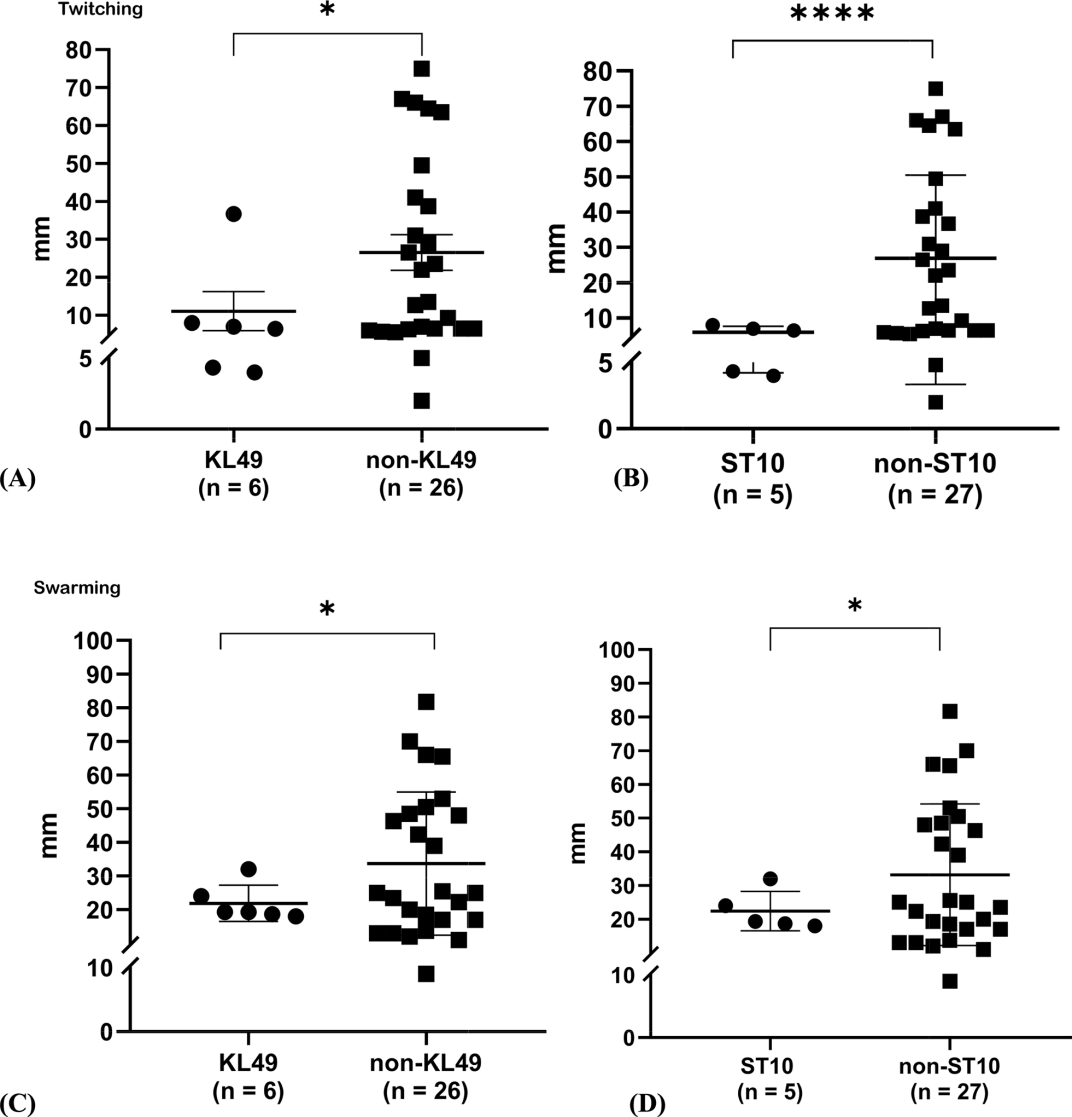
Motility of COAB isolates. Comparison of twitching motility between **A** KL49 and non-KL49 isolates and **B** between ST10^Pas^ and non-ST10^Pas^ isolates**.** Comparison of swarming motility between **C** KL49 and non-KL49 isolates and **D** between ST10^Pas^ and non-ST10^Pas^ isolates. *p*-values were calculated using Welch's t-test (*p < 0.05, ****p < 0.0001)

As motility is associated with the virulence of *A. baumannii* [47, 48], two different motility phenotypes, twitching and swarming motility, were assessed in these strains. Based on the definitions from previous studies [21], our results showed that most of the COAB strains exhibited swarming motility (31/32 strains) and twitching motility (29/32 strains) (Fig. S4A). Although motility varied among the strains, KL49 and ST10^Pas^ strains exhibited significantly lower twitching motility than non-KL49 (Fig. 3A, p = 0.0431) and non-ST10^Pas^ strains (Fig. 3B, p < 0.0001), respectively. A similar trend was observed for swarming motility, with significant differences between KL49 and non-KL49 strains (*p* = 0.0179, Fig. 3C) and between ST10^Pas^ and non-ST10^Pas^ strains (*p* = 0.0349, Fig. 3D). The distributions of twitching and swarming motility among the different KL and ST types are shown in Figures S4B and S4C, respectively. Comparing the COAB and HAAB strains revealed no differences in twitching or swarming motility (Fig. S4D and S4E, respectively).

### Serum resistance of KL49

**Fig. 4:**
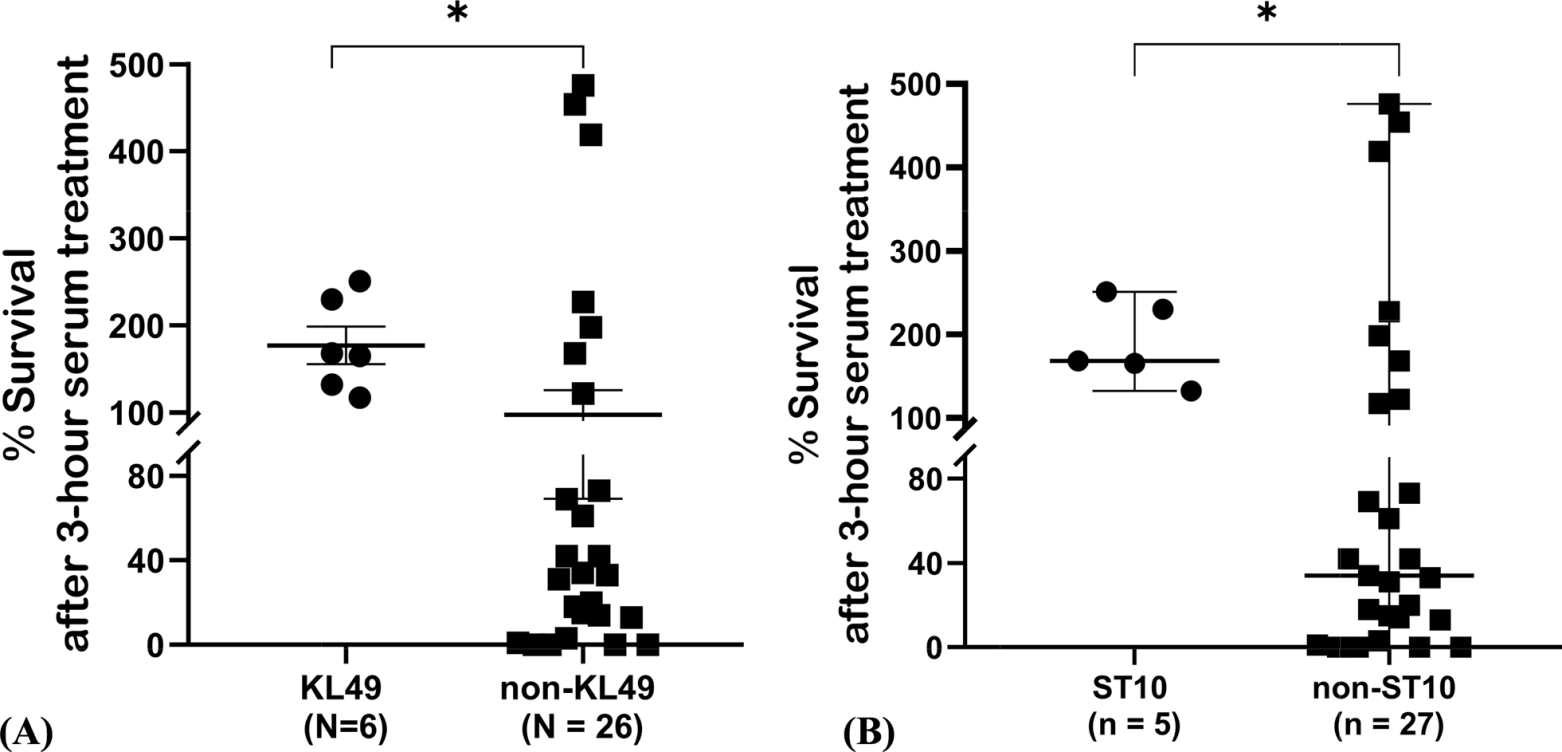
Serum susceptibility of COAB isolates (%) **A** between KL49 and non-KL49 isolates and **B** between ST10^Pas^ and non-ST10^Pas^ isolates. *p*-values were calculated using Welch's t-test (*p < 0.05)

Serum susceptibility is considered a key virulence determinant in *A. baumannii* [14, 49]. Therefore, we examined the survival of the 32 COAB isolates in normal human serum (NHS). After a 3-h treatment, 13 strains exhibited high resistance to serum killing (> 100% survival rate), which not only survived but also could replicate in serum (Fig. S5A). Notably, all six KL49 strains were highly resistant to serum, with survival rates of 117%–251%. The serum susceptibility of KL49 strains was compared with that of non-KL49 strains, and the KL49 strains showed significantly higher resistance (*p* = 0.0350, Fig. 4A). Likewise, ST10^Pas^ strains exhibited significantly higher serum resistance than non-ST10^Pas^ strains (*p* = 0.0178, Fig. 4B). The distributions of serum resistance rates among different KL types and ST types are shown in Figures S5B and S5C, respectively. No significant difference was observed between the COAB and HAAB strains (Fig. S5D).

### Virulence in a *Galleria mellonella* model

**Fig. 5:**
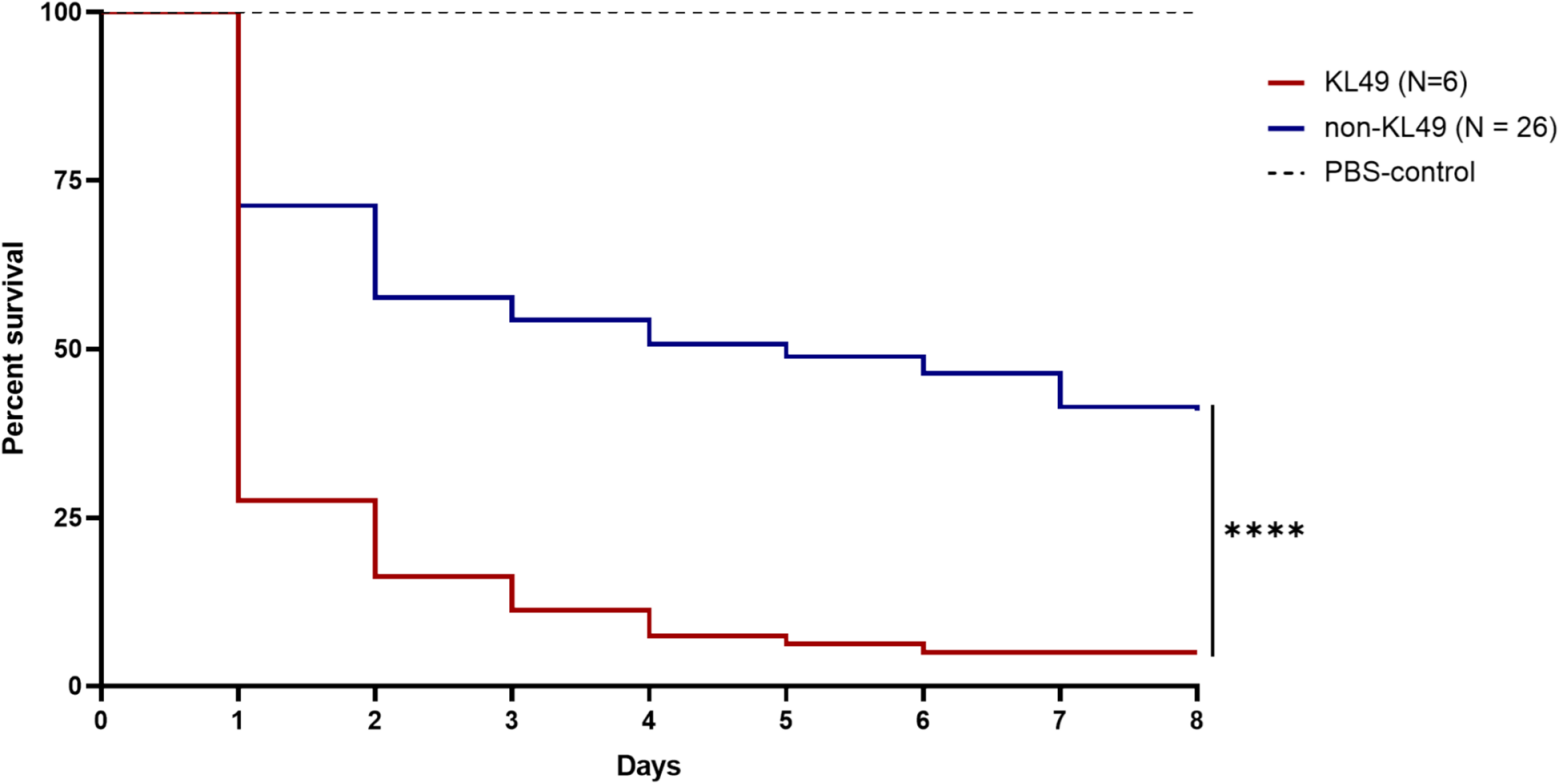
In vivo virulence using a *Galleria mellonella* larvae model. Kaplan–Meier survival curves of *G. mellonella*. This figure presents the survival rates of larvae infected with KL49 and non-KL49 groups, and comparison between KL49 and non-KL49 using the long-rank test revealed significant differences (****p < 0.0001). For the survival rates of individual strains, please refer to Fig. S6A–S6C

The in vivo virulence of the 32 COAB strains was analyzed using *G. mellonella* as a model host. The larvae were inoculated with 5 × 10^5^ CFU of each strain. Previous studies have documented that *A. baumannii* hypervirulent strains generally result in less than 25% larval survival in the *G. mellonella* infection model with an inoculation dose of ~ 10^5^ CFU [50–52]. Here, we found that all KL49 (either ST10^Pas^ or non-ST10^Pas^, *n* = 6), KL9 (ST2^Pas^, ST141^Pas^, and ST866^Pas^, *n* = 3), KL10/ST1459^Pas^ (*n* = 2), KL8/ST1819^Pas^ (*n* = 1), and KL84/ST1336^Pas^ (*n* = 1) strains exhibited survival rates ≤ 25%, whereas the remaining strains, including KL2 (*n* = 3), KL22 (*n* = 2), KL14 (*n* = 3), KL others (*n* = 4), and unknown KL type (*n* = 7), had survival rates ranged from 32 to 100% (Fig. S6A–S6C). Kaplan–Meier survival curves over 8 days of observation are shown in Fig. 5, and log-rank tests indicated that KL49 strains caused significantly lower larval survival than non-KL49 strains (*p* < 0.0001), indicating their hypervirulent phenotype in the *G. mellonella* model. Besides, when comparing ST10^Pas^ and non-ST10^Pas^ strains, a significant difference was also observed (*p* < 0.0001). The survival rates of larvae infected with various KL and ST types are shown in Fig. S6D and S6E, respectively. In addition, no significant difference was observed between the COAB and HAAB strains (Fig. S6F).

### Demographic and clinical factors of the 32 patients infected with COAB according to KL49/non-KL49 or ST10^Pas^/non-ST10^Pas^

As described above, KL49 and ST10^Pas^ strains showed higher virulence in the serum resistance and larvae survival assays. Therefore, we analyzed the clinical records of patients infected with KL49 (*n* = 6) strains versus non-KL49 (*n* = 26) strains and those of patients infected with ST10^Pas^ (*n* = 5) strains versus non-ST10^Pas^ (*n* = 27) strains. Their demographic characteristics, underlying medical conditions, and clinical manifestations are shown in Table 2. No significant differences in age, gender, Charlson scores, and administration of appropriate antimicrobial therapy within 24 h were observed between the patients in these groups (i.e., KL49 versus non-KL49, and ST10^Pas^ versus non-ST10^Pas^). Notably, KL49 and ST10^Pas^ strains appeared to be associated with underlying chronic obstructive pulmonary disease (COPD; *p* = 0.012 and 0.004, respectively), and pneumonia as the source of bacteremia was significantly higher for both KL49 and ST10^Pas^ isolates (*p* = 0.018 and 0.043, respectively). The Pitt bacteremia score was significantly higher in patients infected with KL49 strains than in those infected with non-KL49 strains (*p* = 0.013). A similar trend was observed in patients infected with ST10^Pas^ versus patients infected with non-ST10^Pas^ strains (*p* = 0.003). Specifically, 30-day mortality was higher for patients infected with the KL49 (*p* = 0.039) or ST10^Pas^ strains (*p* = 0.015), suggesting that KL49 and ST10^Pas^ strains of COAB are associated with more severe clinical conditions.

**Table 2:** Demographic data and clinical manifestations of 32 patients with COAB infections

Variables	KL type		p* for comparing KL49 and non-KL49	ST^Pas^ type		p* for comparing ST10 and non-ST10
	KL49 (*n* = 6)	Non-KL49 (*n* = 26)		ST10 (*n* = 5)	Non-ST10 (*n* = 27)	
Age (years), mean (SD)	68.33 (9.07)	68.85 (12.29)	0.924	68.6 (10.11)	68.78 (12.06)	0.976
*Gender, number (%)*	KL49	Non-KL49				
Male	6 (100)	16 (61.54)	0.142	5 (100.0)	17 (62.96)	0.155
Female	0 (0)	10 (38.46)		0 (0.00)	10 (37.04)	
Charlson score, mean (SD)	3.17 (1.60)	4.92 (2.71)	0.140	3.20 (1.79)	4.85 (2.68)	0.199
*Comorbidities, number (%)*						
Hypertension	2 (33.33)	11 (42.31)	1.000	2 (40.00)	11 (40.74)	1.000
Solid malignancies	0 (0.00)	13 (50.00)	0.059	0 (0.00)	13 (48.15)	0.064
COPD	4 (66.67)	3 (11.54)	**0.012**	4 (80.00)	3 (11.11)	**0.004**
Chronic renal insufficiency	2 (33.33)	5 (19.23)	0.590	1 (20.00)	6 (22.22)	1.000
DM with end-organ disease	1 (16.67)	5 (19.23)	1.000	1 (20.00)	5 (18.52)	1.000
Liver cirrhosis	0 (0.00)	6 (23.08)	0.565	0 (0.00)	6 (22.22)	0.555
Tumor without metastasis	0 (0.00)	6 (23.08)	0.565	0 (0.00)	6 (22.22)	0.555
Tumor with metastasis	0 (0.00)	5 (19.23)	0.555	0 (0.00)	5 (18.52)	0.564
Congestive heart failure	0 (0.00)	4 (15.38)	0.566	0 (0.00)	4 (14.81)	1.000
Coronary artery disease	0 (0.00)	1 (3.85)	1.000	0 (0.00)	1 (3.70)	1.000
Autoimmune disease	0 (0.00)	0 (0.00)	·	0 (0.00)	0 (0.00)	·
Leukemia	0 (0.00)	1 (3.85)	1.000	0 (0.00)	1 (3.70)	1.000
Lymphoma	0 (0.00)	1 (3.85)	1.000	0 (0.00)	1 (3.70)	1.000
Use of immunosuppressive agent, number (%)	0 (0.00)	5 (19.23)	0.555	0 (0.00)	5 (18.52)	0.564
24 h appropriate antibiotic treatment, number (%)	4 (66.67)	17 (65.38)	1.000	4 (80.00)	17 (62.96)	0.637
*Source of bacteremia, number (%)*						
Primary bacteremia	0 (0.00)	7 (26.92)	0.296	0 (0.00)	7 (25.93)	0.560
Pneumonia	6 (100.0)	10 (38.46)	**0.018**	5 (100.0)	11 (40.74)	**0.043**
Surgical site infection	0 (0.00)	0 (0.00)	·	0 (0.00)	0 (0.00)	·
Intra-abdominal Infection (IAIs)	0 (0.00)	2 (7.69)	1.000	0 (0.00)	2 (7.41)	1.000
UTI	0 (0.00)	6 (23.08)	0.565	0 (0.00)	6 (22.22)	0.555
Catheter-related infection	0 (0.00)	0 (0.00)	·	0 (0.00)	0 (0.00)	·
Polymicrobial bacteremia, number (%)	0 (0.00)	10 (38.46)	0.142	0 (0.00)	10 (37.04)	0.155
Pitt score, mean (SD)	7.17 (4.49)	2.88 (3.39)	**0.013**	8.20 (4.15)	2.85 (3.32)	**0.003**
ICU, number (%)	2 (33.33)	10 (38.46)	1.000	1 (20.00)	11 (40.74)	0.626
ICU stay days, mean (SD)	3.17 (6.82)	4.27 (8.83)	0.777	0.40 (0.89)	4.74 (9.00)	**0.021**
Total length of stay, mean (SD)	12. (12.51)	14.62 (13.54)	0.669	10.8 (13.59)	14.74 (13.29)	0.548
APACHE II score (if ICU = y), mean (SD)	(*n* = 2)	(*n* = 10)		(*n* = 1)	(*n* = 11)	
	37 (7.07)	29.2 (9.99)	0.326	32.00 (.)	30.37 (10.24)	–
*Outcome*						
30-day mortality, number (%)	4 (66.67)	5 (19.23)	**0.039**	4 (80.00)	5 (18.52)	**0.015**

Welch's t-test or Fisher’s exact test was used to assess differences between two groups. Statistically significant results (*p* < 0.05) are indicated in bold

COAB, community-onset *A. baumannii*; COPD, chronic obstructive pulmonary disease; DM, diabetes mellitus; UTI, urinary tract infection; IAIs, intra-abdominal infections

## Discussion

*A. baumannii* is a significant public health concern not only within hospital environments, but also in the community, where environmental and animal reservoirs may serve as potential sources and contribute to the circulation and emergence of CAAB/COAB strains [9, 53, 54]. Cases of CAAB/COAB are more frequently reported in tropical and sub-tropical climates, such as northern Australia [4, 55], Taiwan [26, 56], Korea [57], Hong Kong [6], China [11], and Singapore [8], whereas in more temperate climates, like Europe [58] and America [59, 60], cases are rare. Although multidrug resistance in *A. baumannii* is less common among community isolates than among hospital isolates, resistance to the third-generation antibiotics (cephalosporins and fluoroquinolones) used to treat community-acquired pneumonia (CAP) has been documented among community *A. baumannii* isolates [4, 25]. A study in China found that 15 of 32 *A. baumannii* isolates causing CAP were resistant to three or more antimicrobial classes [11]. A study conducted in Turkey reported significantly increased resistance to gentamicin, ertapenem, levofloxacin, and trimethoprim–sulfamethoxazole among CAAB in the post-COVID-19 pandemic period [61]. In contrast, a study conducted in Northern Australia reported that all tested COAB isolates were susceptible to gentamicin and meropenem, indicating that not all community-acquired strains are multidrug-resistant, and such differences may be due to variation in the study regions [10]. In our current study, consistent with previous findings, the resistance rates of COAB strains were significantly lower than those of HAAB strains for all tested antibiotics, except for colistin, for which no significant difference was observed (Table S3). The lower antibiotic resistance rates to meropenem and other agents in COAB compared with HAAB may provide important guidance for empiric therapy in clinical settings in Taiwan. However, since the number and sources of COAB strains were limited (collected from only two hospitals), further studies are needed to strengthen these implications. Notably, non-susceptible COAB strains were identified for ceftazidime (12.5%), cefepime (12.5%), ciprofloxacin (12.5%), gentamicin (12.5%), and meropenem (3.1%), all of which have been suggested for treating CAAB/COAB infections [26]. In addition, one strain (3.1%) was identified as multidrug-resistant (MDR). Despite the low rate of MDR in our isolates, resistance rates to certain antibiotics were higher than those reported in a previous study of strains collected in Taiwan between 1994 and 1999 [26], including ceftazidime (12.5% vs. 7.1%), cefepime (12.5% vs. 7.1%), and carbapenem (0% vs. 3.1%), suggesting a continuous increase in antibiotic resistance among CAAB/COAB isolates.

It has been suggested that different clinical settings are associated with distinct KL types [20, 21, 62, 63], and some KL types may exhibit higher virulence [16, 64, 65]. For example, HAAB was typically associated with types KL2, KL3, and KL22 [16, 32]. In addition, some unique KL types were reported in specific geographic regions, such as KL58 in Vietnam [66], KL1 in Afghanistan [67], KL17 in Israel [42], and KL52 in Taiwan [20]. In contrast, information about CAAB/COAB is relatively limited. Only one study from Australia analyzed the KL types of COAB; they found that KL49 was the most common KL type (58.5%) (in this study, KL49 was also common in HAAB), while the remaining COAB isolates were of diverse capsule types [10]. Here, we found that the KL type distributions among HAAB and COAB isolates in Taiwan are distinct; KL49 was identified as the major KL type among COAB, whereas KL2, KL10, KL22, and KL52 accounted for the majority of HAAB isolates. This suggests that the two populations of strains develop distinct properties to adapt to different environments.

Our study identified ST10 (Pasteur scheme) as the most prevalent ST type and showed that it was strongly associated with KL49 strains. However, the remaining isolates (non-KL49/ST10^Pas^) were of diverse KL and ST types. In Vietnam, both community-acquired and hospital-acquired ST10^Pas^ strains have been documented [66]. This ST type was associated with an outbreak of extensively drug-resistant HAAB in the USA [45], and was also identified as a predominant type among community-onset strains in Australia [10]. Based on a previous analysis, the most recent common ancestor of ST10^Pas^ was estimated to have emerged in 1626–1826, with evidence of multiple introduction events in Australia and Southeast Asia over time [10]. These findings highlight the broad geographical spread of ST10^Pas^ and its ability to colonize and cause disease. Furthermore, ST10^Pas^ strains are thought to be more virulent than other strains, as supported by various lines of evidence such as the high mortality rate observed during an outbreak in the USA among relatively immunocompetent individuals [45] and the demonstration of hypervirulence in mouse models of infection [68]. Our study, which documented ST10^Pas^-associated KL49 *A. baumannii* in the community for the first time in Taiwan, indicates that the global dissemination of ST10^Pas^ is worthy of great attention.

ICs, designated based on genetic diversity, have been used to examine the dissemination of various *A. baumannii* clones across different geographical regions. To date, nine ICs (IC1–IC9) have been documented [37], with IC1–IC3 most likely playing contributory roles in the global dissemination of *A. baumannii* infections, whereas IC4–IC9 are considered to be associated with certain countries or continents and are less well studied [69–72]. IC8 has been reported in isolated case reports from several countries, such as India [73], Afghanistan [74], Bangladesh [75], and China [76], and represents a large fraction of isolates reported in Europe [77, 78] and Australia [10]. In a surveillance study of CRAB isolates from nine regions in Asia, including Taiwan, clonal complex (CC) 92, corresponding to IC2, was identified as the most prevalent, accounting for 76.9% (83/108) of isolates [79]. In 2020, a case report from the USA identified a rare instance of CAP and bloodstream infection caused by a multidrug-resistant *A. baumannii* (MDRAB) strain belonging to clone ST451^Oxf^ (part of IC2), which is prevalent in Asia. This highlights the potential for transnational transmission of this CAAB clone [60]. Our study identified IC8 (*n* = 5), composed exclusively of KL49/ST10^Pas^ strains, as the most predominant IC type among the COAB isolates, which was reported here for the first time in Taiwan and provides further evidence of potential cross-country transmission within community settings. Among 32 COAB, a total of 29 isolates—including 24 strains not assigned to known international clones (IC1-IC9) and 5 IC8 strains (KL49/ST10^Pas^)—did not belong to the major global IC1–IC3 lineages typically associated with nosocomial infections. This finding underscores the genetic heterogeneity of community strains and suggests the possible presence of novel or region-specific clones circulating in Taiwan. WGS will be needed to provide deeper insights into the population structure of these isolates, including confirmation of the clonal relatedness among KL49/ST10^Pas^ strains and identification of putative virulence or resistance-associated genes.

Variations in virulence levels among different KL types have been documented. For instance, (1) Talyansky et al. reported that a loss-of-function in *gtr*6 in KL3 and KL22 isolates of *A. baumannii* altered the CPS structure and reduced virulence by inhibiting complement deposition and phagocyte recognition [80]. (2) A KL1 isolate showed optimal growth in human ascites fluid and survival in both human serum and a rat soft tissue infection model [81]. (3) A study conducted in Taiwan showed higher morbidity and mortality rates for patients infected with four predominant KL types (KL2, KL10, KL22, and KL52) than those for patients infected with other KL types [20]. Here, we revealed the predominance of KL49 among COAB in Taiwan and demonstrated their hypervirulent phenotypes in both in vitro and in vivo models, as they showed significantly higher serum resistance and greater mortality in the *G. mellonella* larvae infection model. Most interestingly, we found that two different ST types of KL49 (KL49/ST10^Pas^ and KL49/ST1017^Pas^) both showed hypervirulent phenotypes, suggesting that KL49 may contribute to virulence. This observation aligns with other studies showing the virulence of KL49/ST2. A study from China reported that KL49/ST2 nosocomial strains were associated with mortality and a hypervirulent phenotype in a larval model [64]. Kai Zhou et al. also showed that Oxford ST457 (representing Pasteur ST2 and IC2) strains carrying KL49 exhibited higher mortality rates and greater virulence in a *G. mellonella* infection model than non-KL49 strains [82]. These studies highlight the significance of KL49 even across various ST types.

Type KL49 capsular polysaccharide contains repeating units of α-8-epi-legionaminic acid, α-L-fucosamine, and α-D-glucosamine [65]. Previous studies showed that capsules containing sialic acids protect Gram-negative bacteria from complement activation [83], and the presence of α-8-epi-legionaminic acid, a sialic acid analog, may contribute to the serum resistance of KL49 strains [21]. However, whether the structure of the KL49 capsule is critical for virulence or if other virulence determinants are associated with KL49 strains remains to be determined. Besides, in our dataset, KL49 and ST10^Pas^ are highly associated, as all ST10^Pas^ isolates belong to KL49. To our knowledge, ST10^Pas^ strains with non-KL49 capsules have not been clearly reported, and thus their independent effects cannot be distinguished. Nonetheless, we cannot exclude the possibility that the ST10^Pas^ background (beyond the capsule locus) also contributes to hypervirulence, suggesting that the KL49/ST10^Pas^ combination could be particularly concerning.

We found that HAAB strains exhibited higher levels of biofilm formation than COAB strains, which is consistent with previous studies of other pathogens [84, 85]. Previous research also showed that biofilm-forming strains survived significantly longer on dry surfaces than non-biofilm-forming strains [86] and that biofilm formation in HAAB isolates significantly enhanced resistance to both desiccation and disinfection by benzalkonium chloride [17]. Interestingly, we observed that although COAB strains produced less biofilm, they exhibited greater resistance to desiccation than HAAB strains. Whether COAB isolates possess certain unique factors that contribute to their tolerance of dry conditions remains to be clarified.

Motility is considered a contributor to bacterial pathogenesis [48]. *A. baumannii* exhibits two distinct modes of movement: twitching [87] and swarming [47] motility. A study by Pérez-Varela et al. found that an *A. baumannii* mutant with reduced surface motility showed decreased virulence in a *Caenorhabditis elegans* infection model [88]. However, contrary to this finding, we observed that although KL49/ST10^Pas^ isolates showed lower motility in both swarming and twitching assays than non-KL49/ST10^Pas^ strains, KL49/ST10^Pas^ isolates displayed hypervirulence. This suggests that motility is not a critical virulence factor for KL49/ST10^Pas^; rather, serum resistance may play a more pivotal role [88–90].

The ability of *A. baumannii* to resist the bactericidal effects of serum is a critical determinant of virulence and the capacity to cause invasive infections [14, 49, 91]. In the current study, variability in serum resistance was observed among the COAB isolates, although KL49 and ST10^Pas^ strains showed significantly higher serum resistance than other capsular and ST types, which was concordant with their hypervirulence in the larvae model. In addition, KL49 and ST10^Pas^ COAB strains were associated with pneumonia and underlying COPD and worse clinical outcomes, including higher mortality rates and significantly elevated Pitt bacteremia scores, underscoring the severe impact of these strains on patient health, which echoes our experimental data. While our findings revealed a strong association between KL49/ST10^Pas^ strains and severe clinical outcomes, there could be other unmeasured confounding factors. For example, smoking and chronic alcohol use—both recognized as risk factors for community-acquired infections [26, 55, 92]—were not recorded in our dataset. Therefore, further studies incorporating detailed patient lifestyle and comorbidity data are needed to clarify causality.

This study has several limitations. First, the small sample size limited the statistical power (Table S4), and therefore, the observed associations should be interpreted as trends rather than definitive conclusions. Second, although multiple genotyping methods were applied to classify major COAB clones (KL49/ST10^Pas^), WGS was not performed, which restricted a more comprehensive analysis of clonal relatedness, virulence determinants, and resistance genes. Third, while we analyzed the virulence traits and clinical records of KL49 and non-KL49 strains, the KL types of seven non-KL49 strains could not be determined using *wzc/wzy* analysis. WGS may be required to resolve their KL types, and further studies are needed to clarify whether these untypable strains are associated with specific virulence patterns or clinical severity. Fourth, we defined community-onset bacteremia as the presence of at least one positive blood culture obtained from a patient with signs and symptoms of infection within 48 h after admission. However, because information on recent healthcare-associated exposures was not available, we were unable to determine whether these cases were truly community-acquired. Therefore, future studies should clarify healthcare contact history to distinguish between community-onset and community-acquired infections. Fifth, although ST10^Pas^ and KL49 genotyping could be useful for clinical detection, their utility in routine clinical care remains limited. In practice, genotyping results are typically not made available in real time, the assays are not standardized across laboratories, and they are not routinely integrated into clinical decision-making. These factors highlight the practical limitations of rapidly identifying KL types or ST types in clinical settings. Finally, since the isolates were collected from only two hospitals in Taiwan, the findings may not fully represent the broader epidemiological spectrum of *A. baumannii* in other regions. In addition, our study focused exclusively on bloodstream infections. Further investigations incorporating both bacteremic and non-bacteremic COAB infections (particularly community-onset pneumonia) are warranted to better characterize the full epidemiology of COAB in Taiwan. Future studies with larger sample sizes, multicenter cohorts, inclusion of diverse disease types, and integrated genomic approaches are needed to monitor, validate, and extend these findings.

## Conclusions

This is the first report identifying KL49/ST10^Pas^ as the predominant clone among COAB isolates in Taiwan. Notably, we demonstrated its hypervirulence using serum bactericidal tests and an in vivo larvae model, and its association with severe clinical outcomes. Early identification of a genetic background linked to hypervirulence may help guide clinicians in implementing timely and appropriate antimicrobial therapy, which may improve outcomes for patients with COAB infections. Although in our COAB collection, the antibiotic resistance rate was relatively low, the rates were slightly higher than those reported in previous studies. Thus, continuous surveillance and monitoring of antibiotic resistance in this emergent hypervirulent clone are urgently needed. To our knowledge, this is the first study in Taiwan to examine the clinical manifestations of *A. baumannii*-associated community-onset bloodstream infections and the molecular epidemiology and virulence traits of COAB isolates. This study establishes a crucial foundation for understanding the molecular epidemiology of COAB infections in Taiwan and possible directions for future research on virulence mechanisms and disease control strategies.

## Supplementary Information

Additional file1 (DOCX 13289 KB)

Additional file2 (DOCX 35 KB)

## Data Availability

All data generated or analyzed during this study are included in this published article and its supplementary information files.

## Abbreviations

COAB: Community-onset *A. baumannii*
CAAB: Community-acquired *A. baumannii*
HAAB: Hospital-acquired *A. baumannii*
KLs: Capsular types
STs: Sequence types
ICs: International clones
LB: Luria–Bertani
CRAB: Carbapenem-resistant *A. baumannii*
MDRAB: Multidrug-resistant *A. baumannii*
