# Supplementary material for: Emerging virulent clones of community-onset Acinetobacter baumannii in Taiwan
Source: Trop Med Health. 2025 Dec 16;53:189. doi: 10.1186/s41182-025-00850-1 (PMC12709753; doi:10.1186/s41182-025-00850-1)
Supplement: Supplementary file 1 — Additional file1 (DOCX 13289 KB) [file 41182_2025_850_MOESM1_ESM.docx]

**
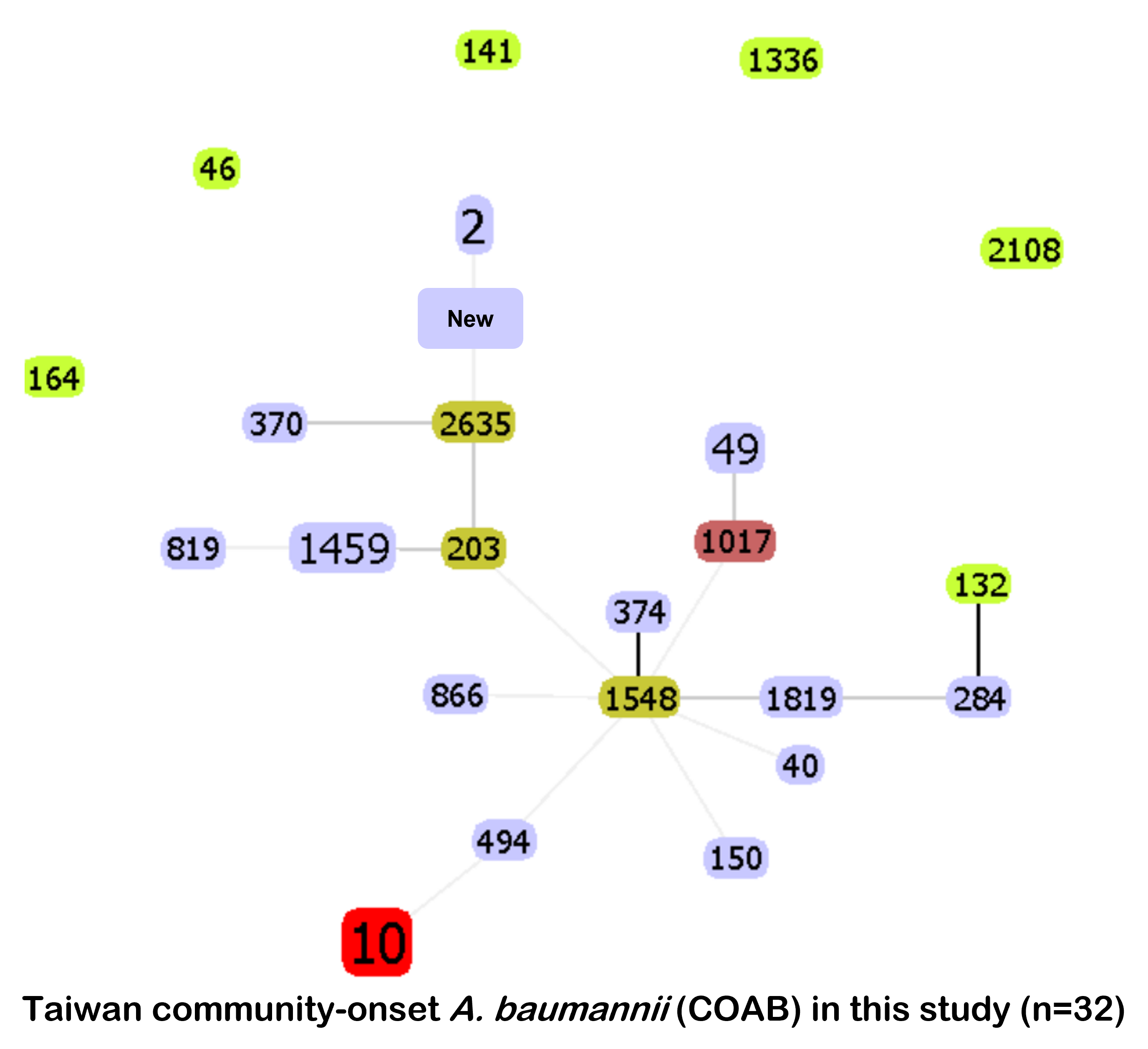
(A)**

**
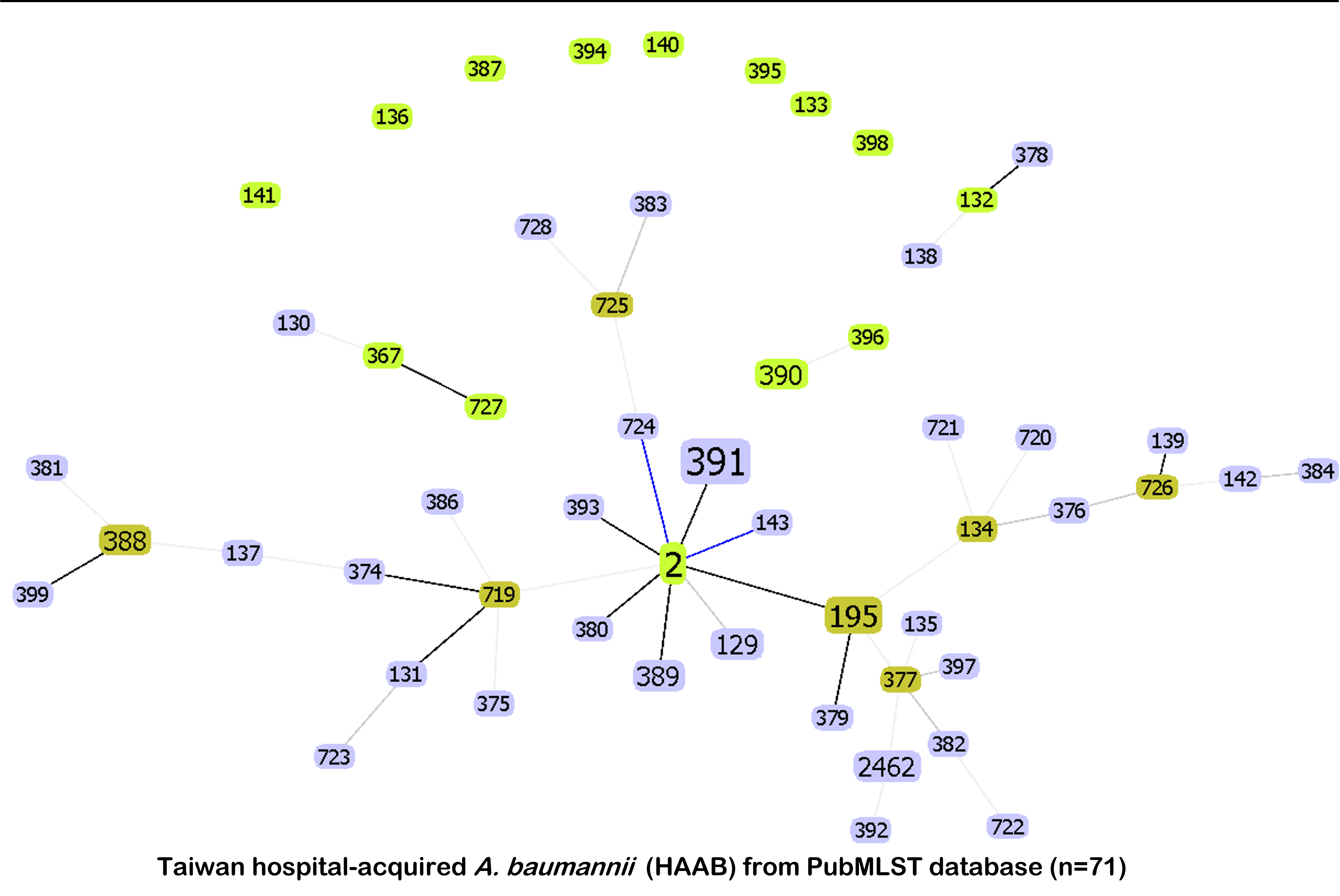
(B)**

**FIG S1 ST-Based clustering of COAB strains using goeBURST (global optimal Based Upon Related Sequence Types)**. Pasteur scheme, **(A)** The relatedness of the 32 COAB isolates in this study. **(B)** The relatedness of 71 HAAB isolates from the PubMLST database. ST Node Colors are as follows: Light green for group founders, dark green for sub-group founders, light blue for common nodes, and ruby red and bright red for highlighted nodes of the major KL type, KL49. Link Colors are as follows: Both black and blue links show Single Locus Variants (SLVs), darker gray links show Double Locus Variants (DLVs), and lighter gray links show Triple Locus Variants (TLVs). The detailed color conventions are described in the Materials and Methods.

**
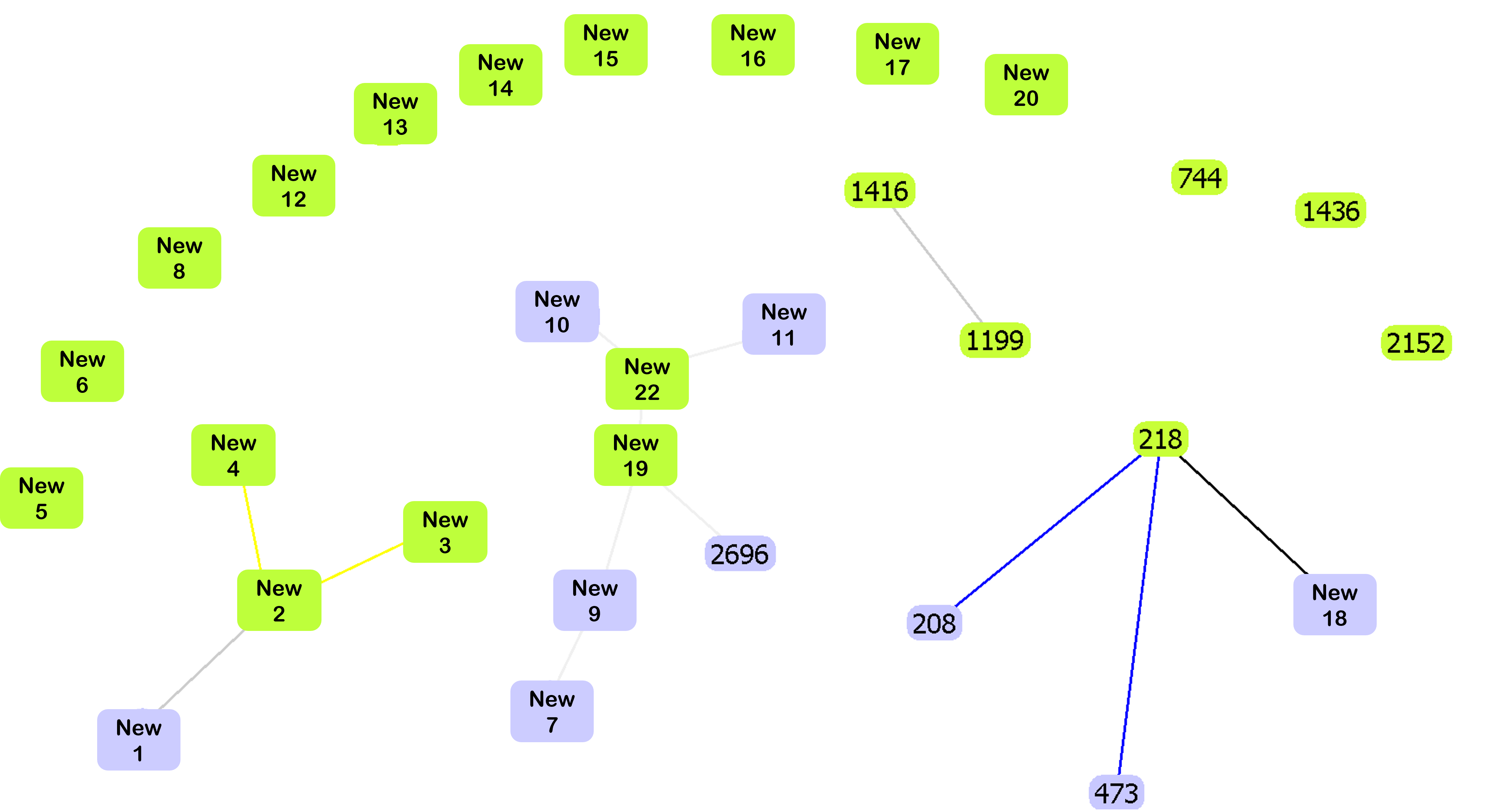
(C)**

**FIG S1 Continued. (C)** The relatedness of STs (Oxford scheme) for the 32 COAB isolates in this study. Link Colors are as follows: Both black and blue links show SLVs, darker gray links show DLVs, lighter gray links show TLVs, and yellow links indicate connections resolved using tiebreak rules. The detailed color conventions are described in the Materials and Methods.

**(A)**


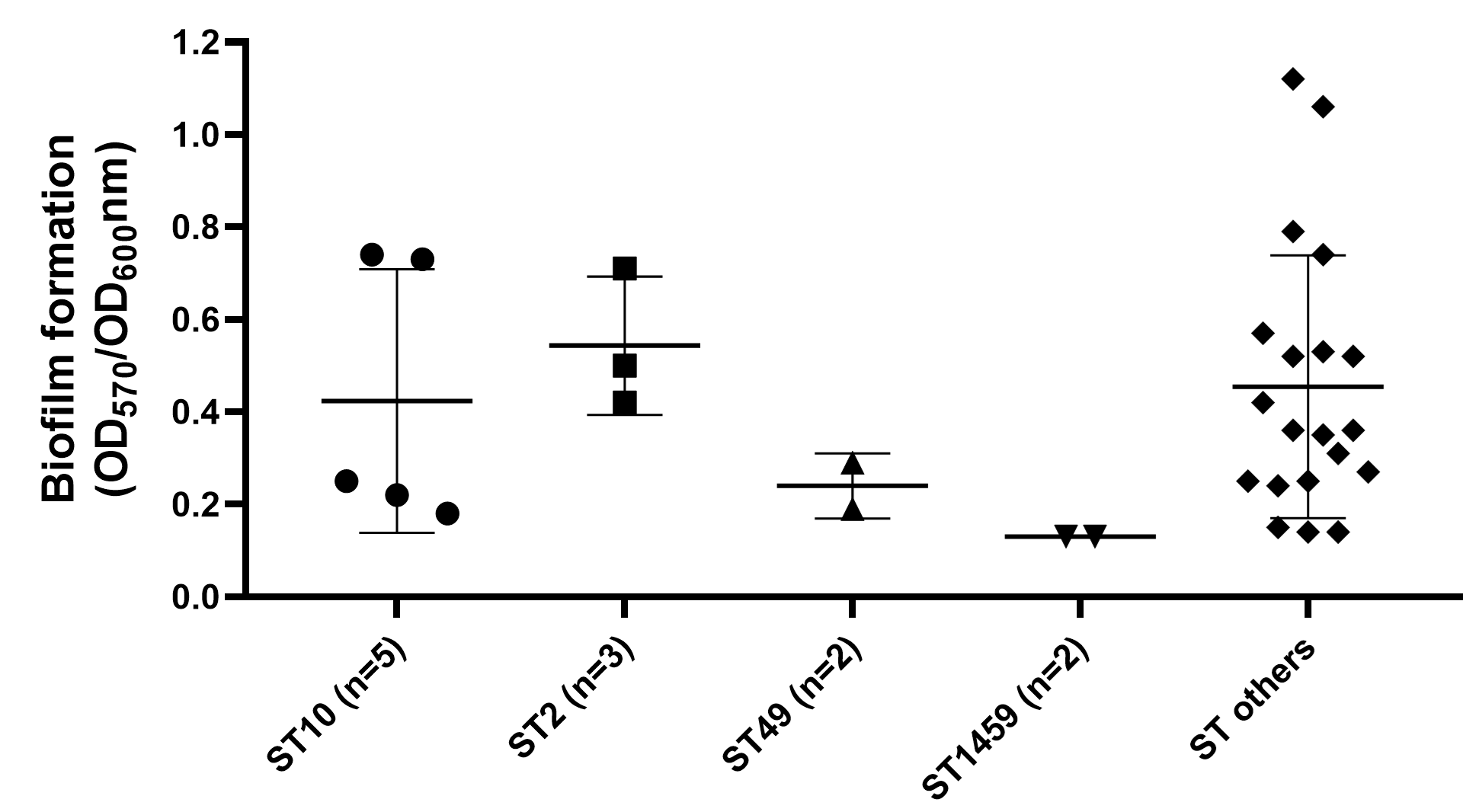
**
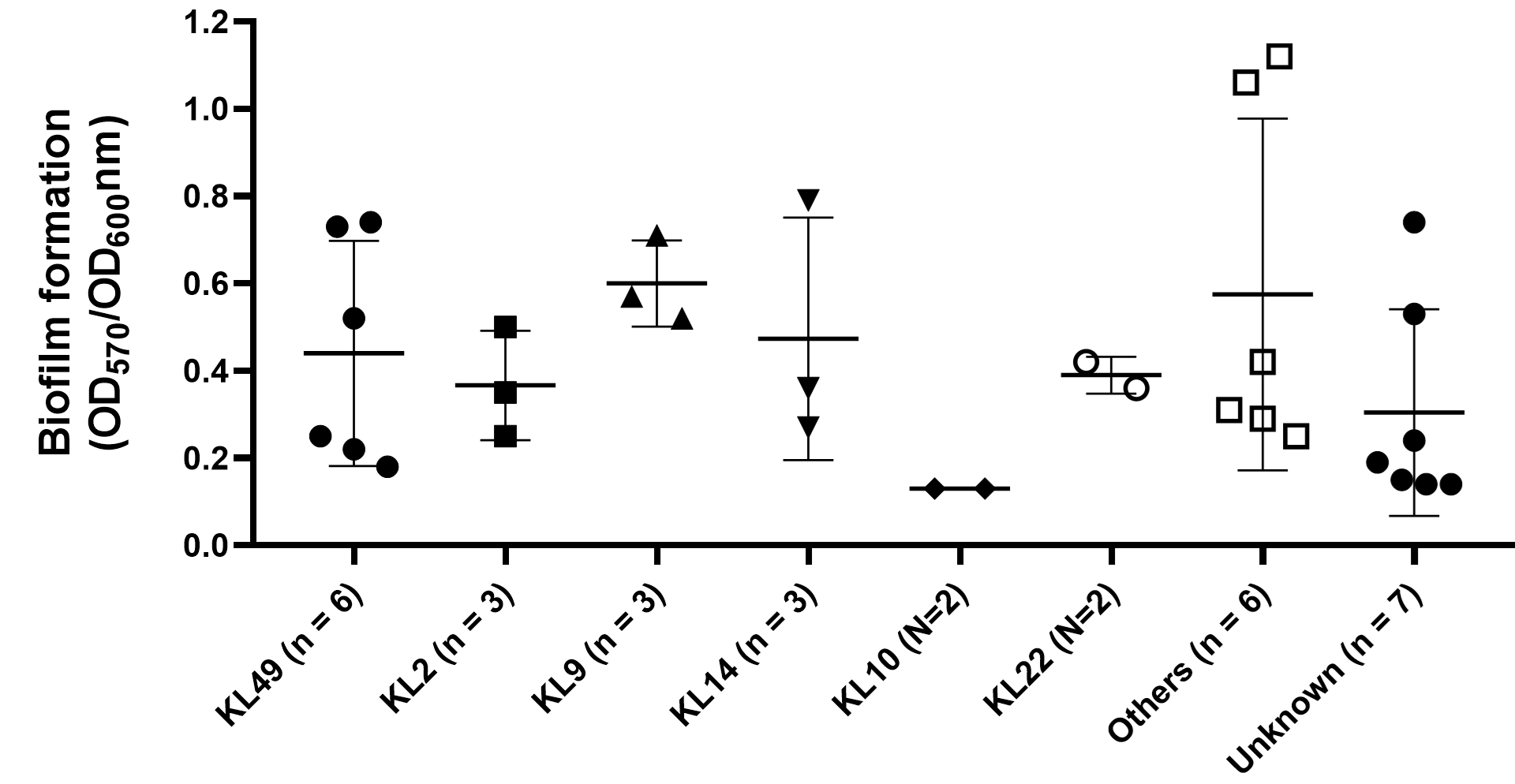
**

**(C)**


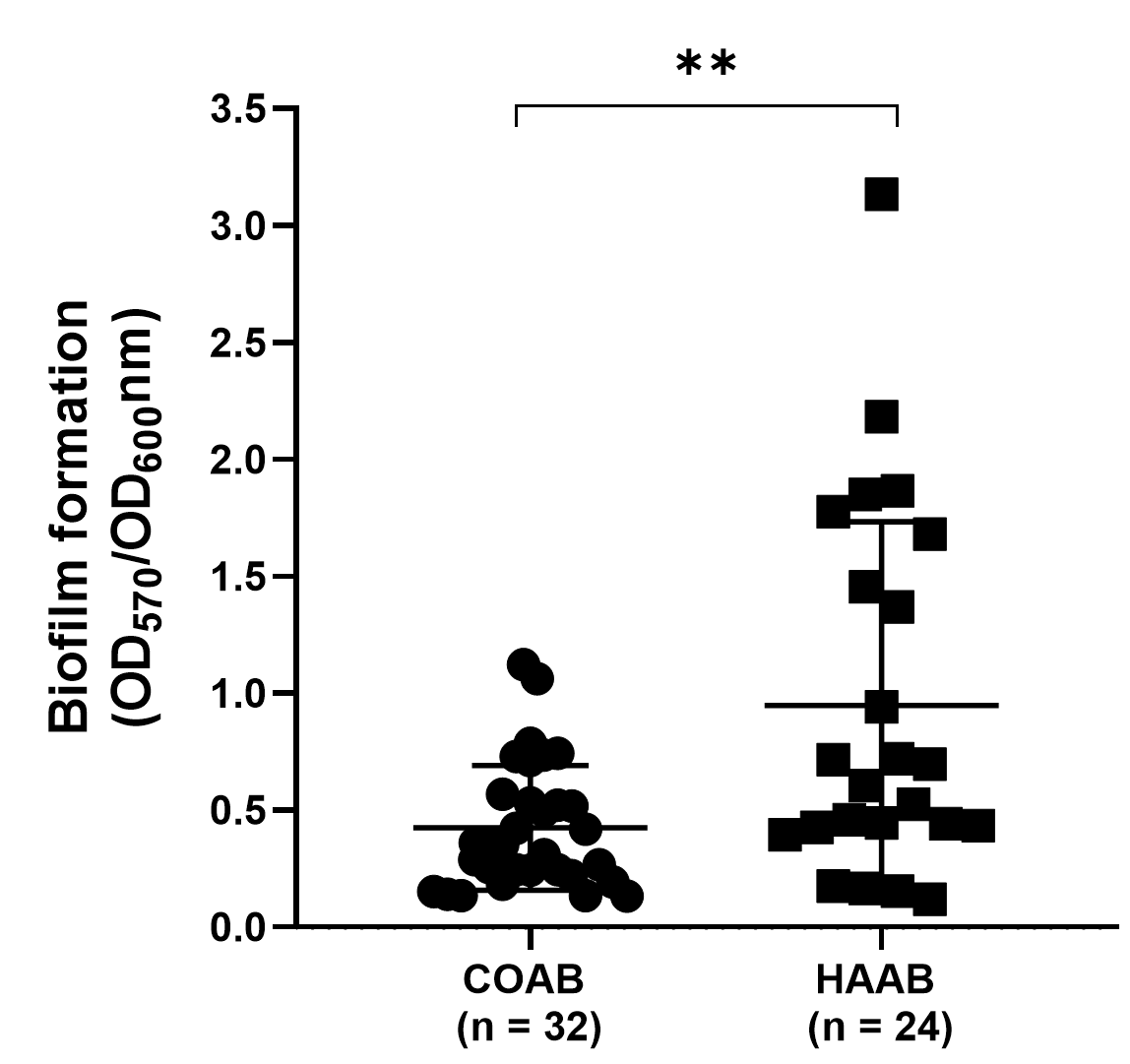


**(B)**

**FIG. S2 Biofilm formation ability of the 32 COAB strains.** Distribution of the biofilm formation for different **(A)** KL types and **(B)** ST types. **(C)** Comparison of biofilm formation between COAB and HAAB isolates. The *P-*value was calculated using Student's t-test (***p* < 0.01).

**
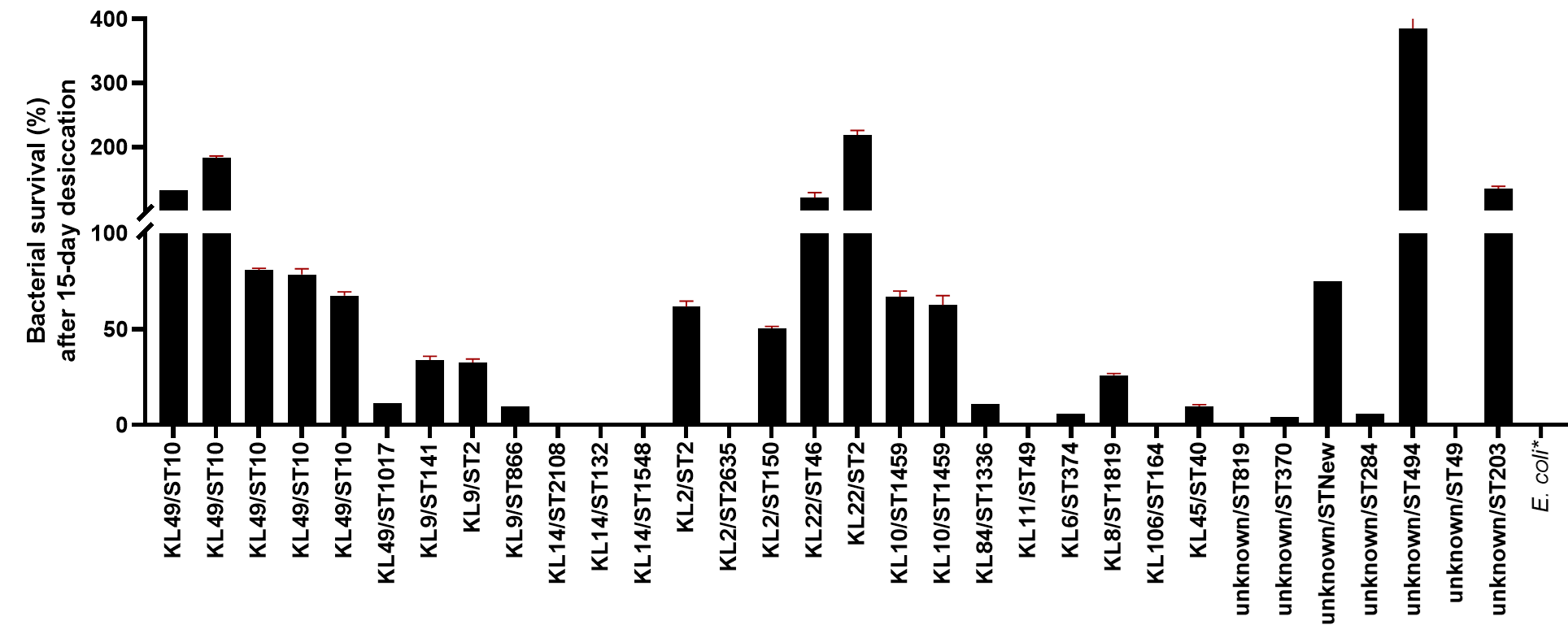
(A)**

**
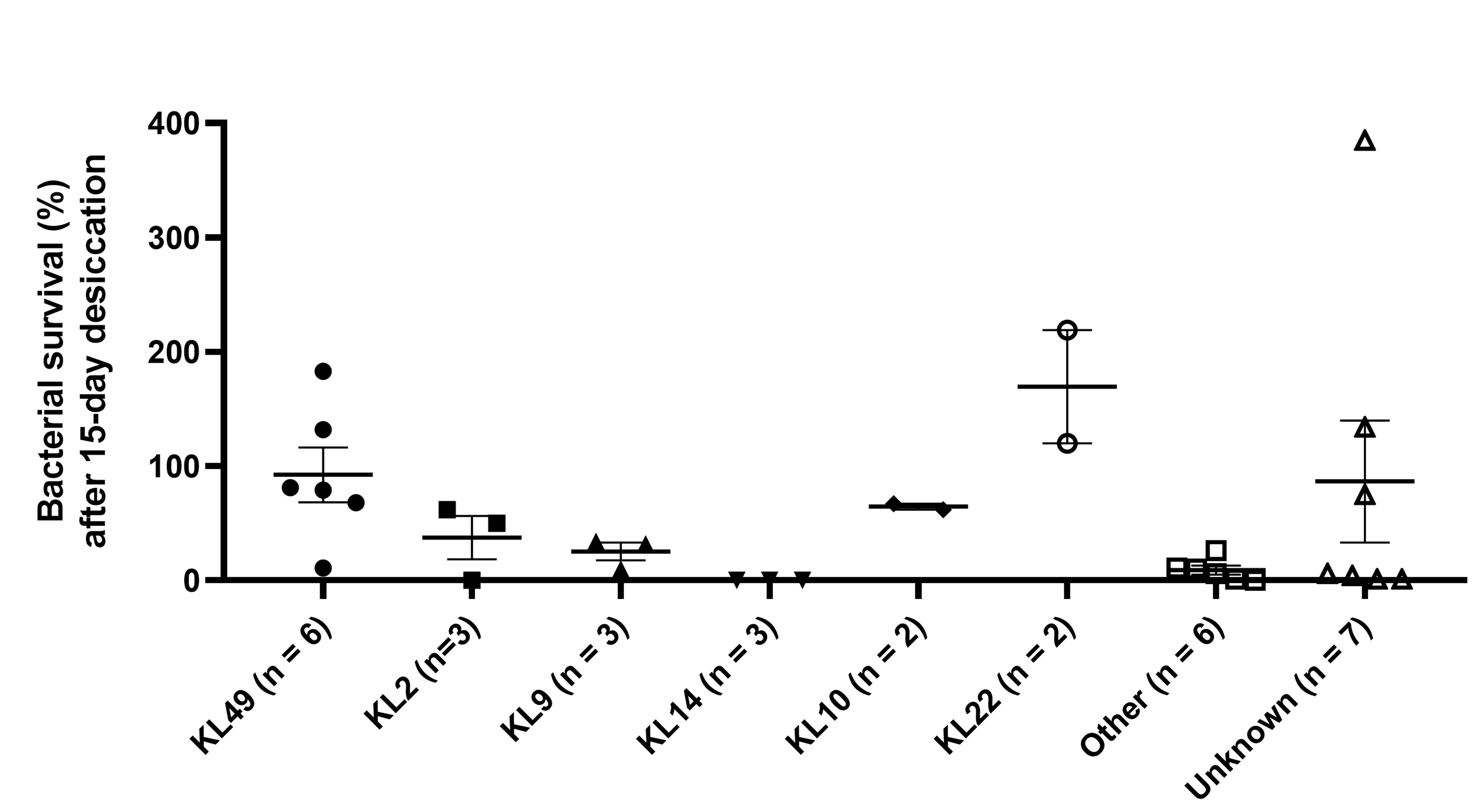
(B)**


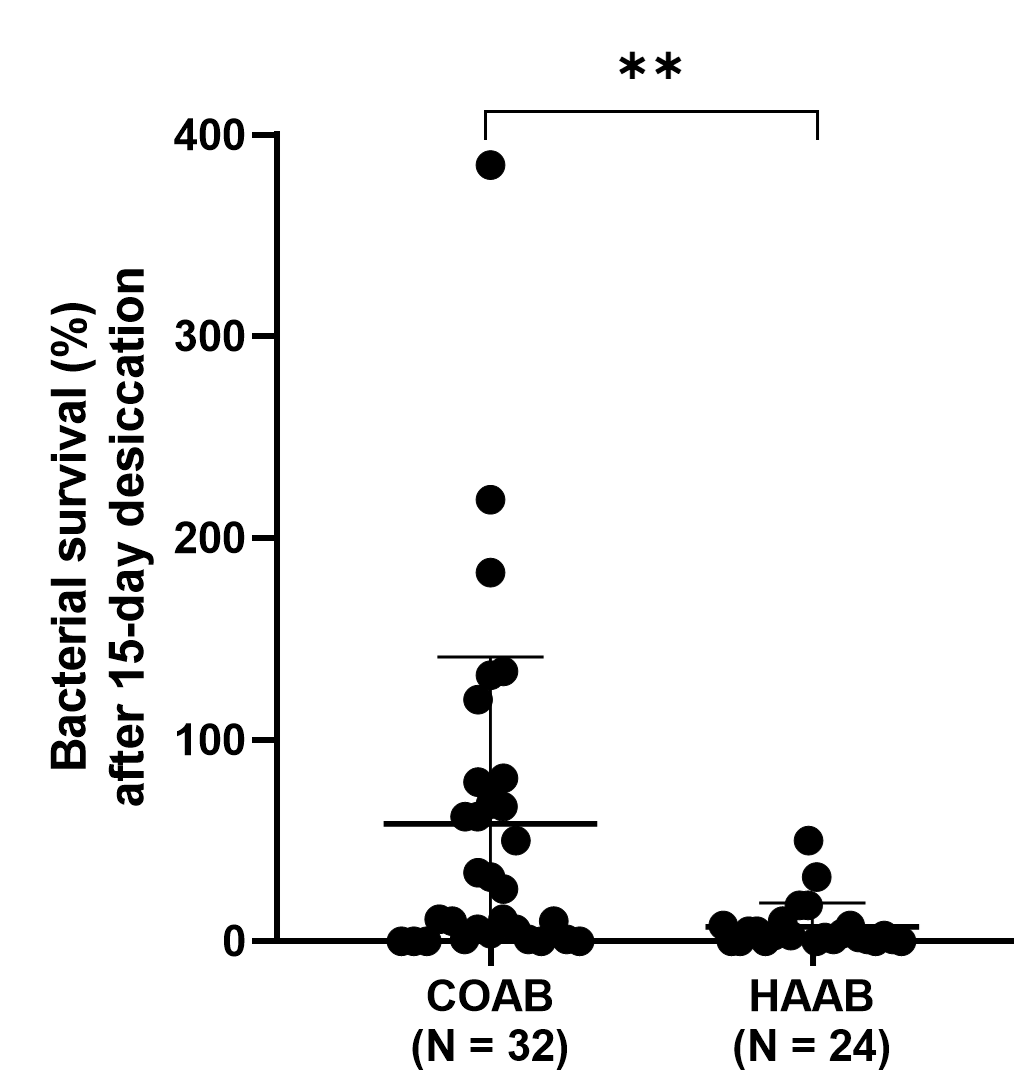
 **(D)**


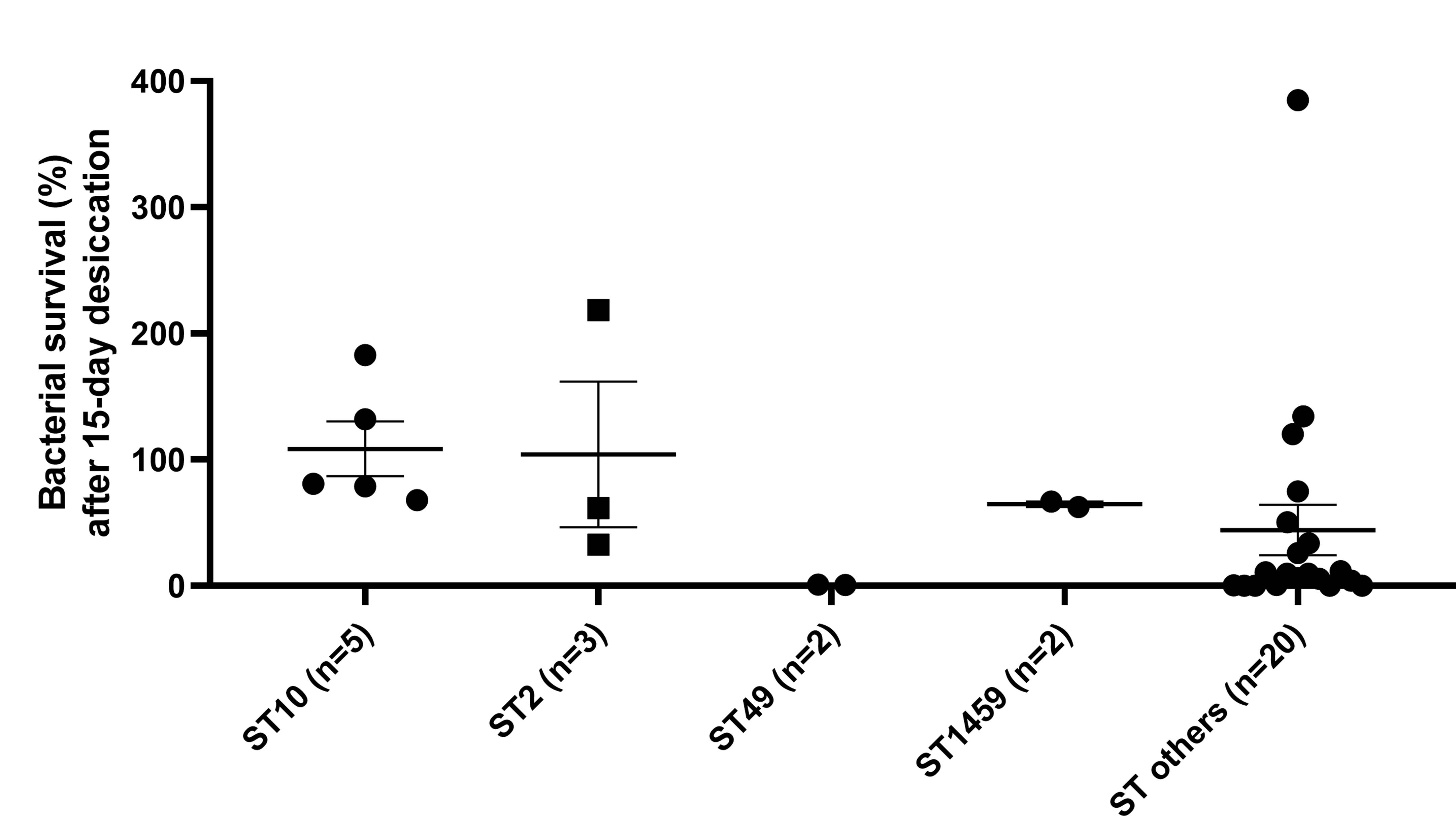
**(C)**

**FIG. S3 The desiccation survival of the 32 COAB strains. (A)** The survival percentage of the 32 COAB strains after 15 days of desiccation**.** Distributions of resistance rate for different **(B)** KL types and **(C)** ST types. **(D)** Comparison of bacterial survival percentage between COAB and HAAB isolates. The *P-*value was calculated using Student's t-test (**p < 0.01).

**
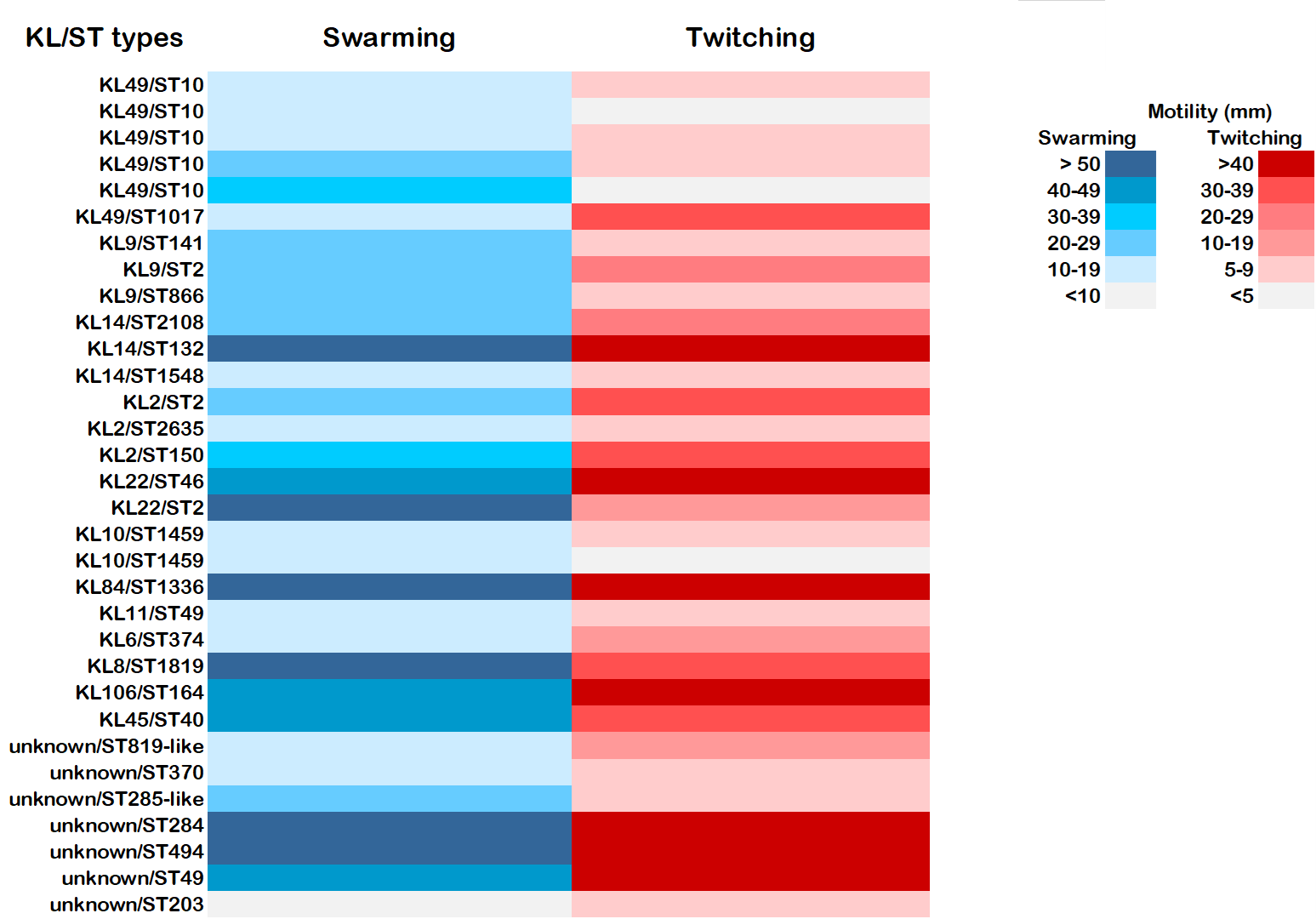
(A)**

**
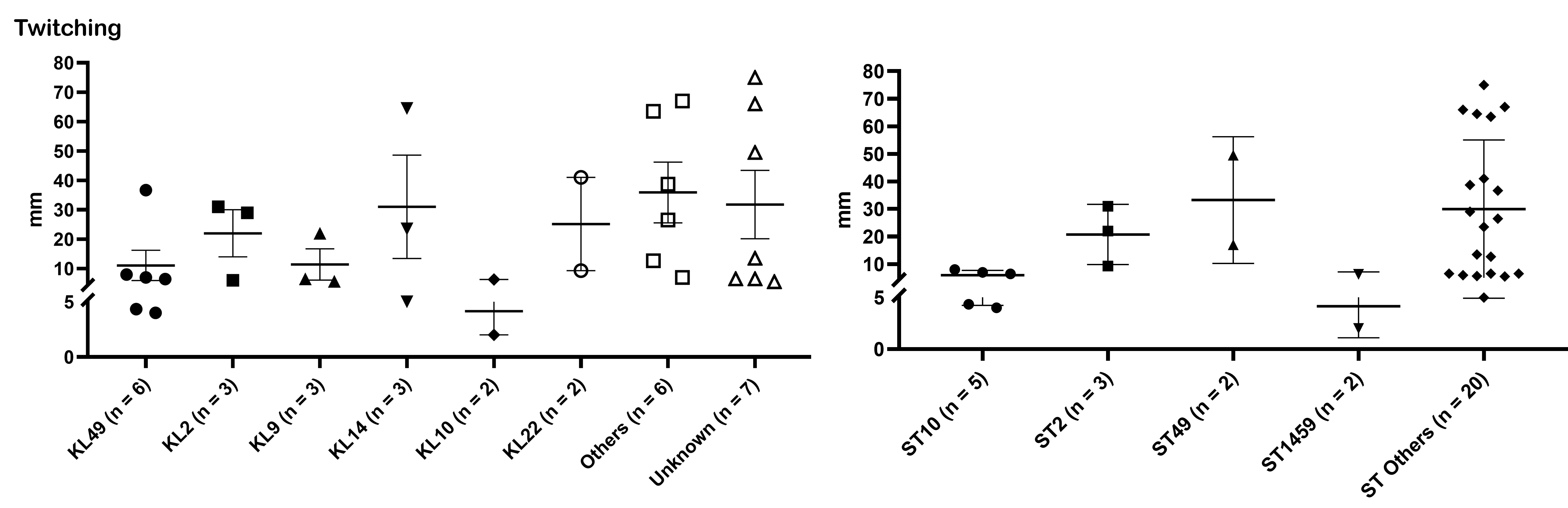
**

**(B)**

**
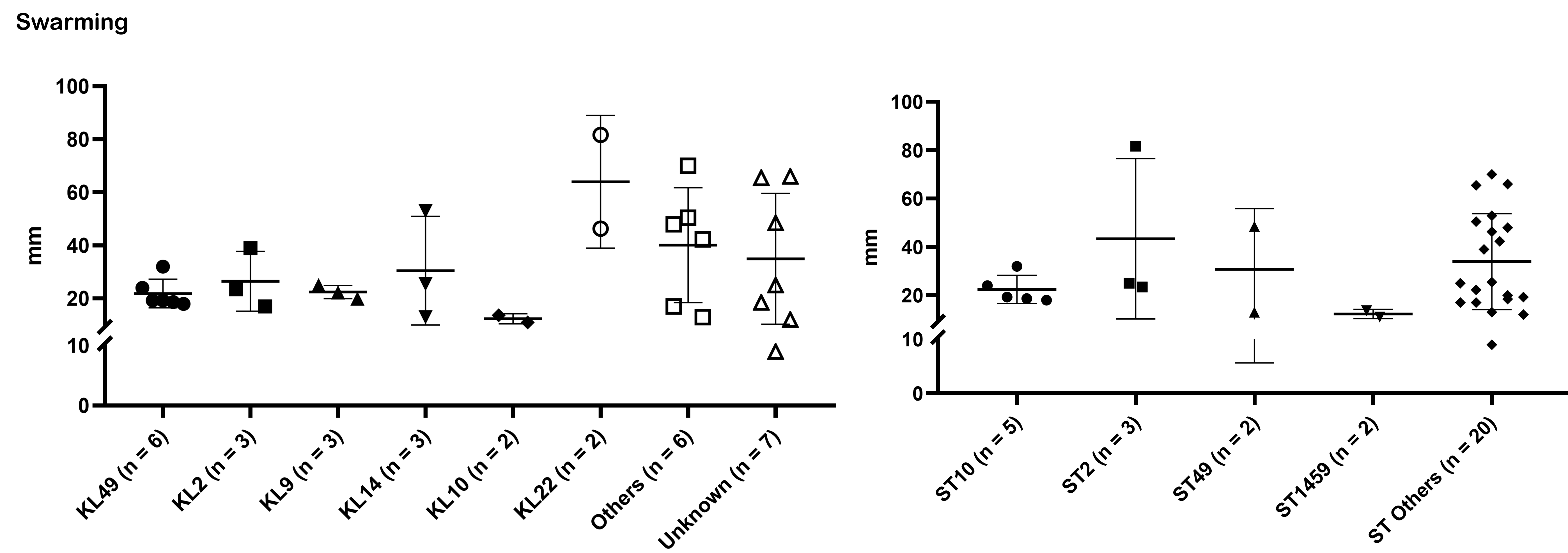
**

**(C)**

**FIG. S4 Motility properties of the COAB isolates. (A)** Motility phenotypes of the 32 isolates**. (B)** Distribution of twitching motility for the various KL and ST types. **(C)** Distribution of swarming motility for the various KL and ST types.

**
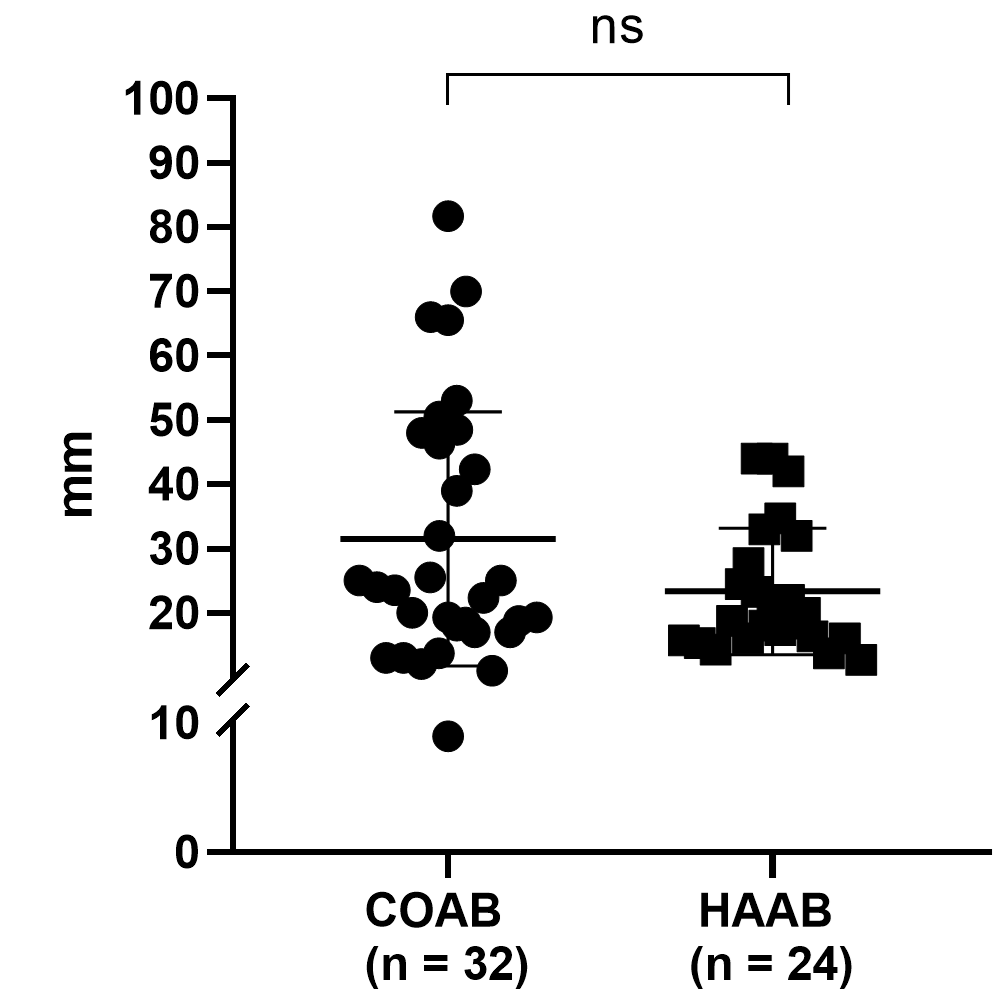

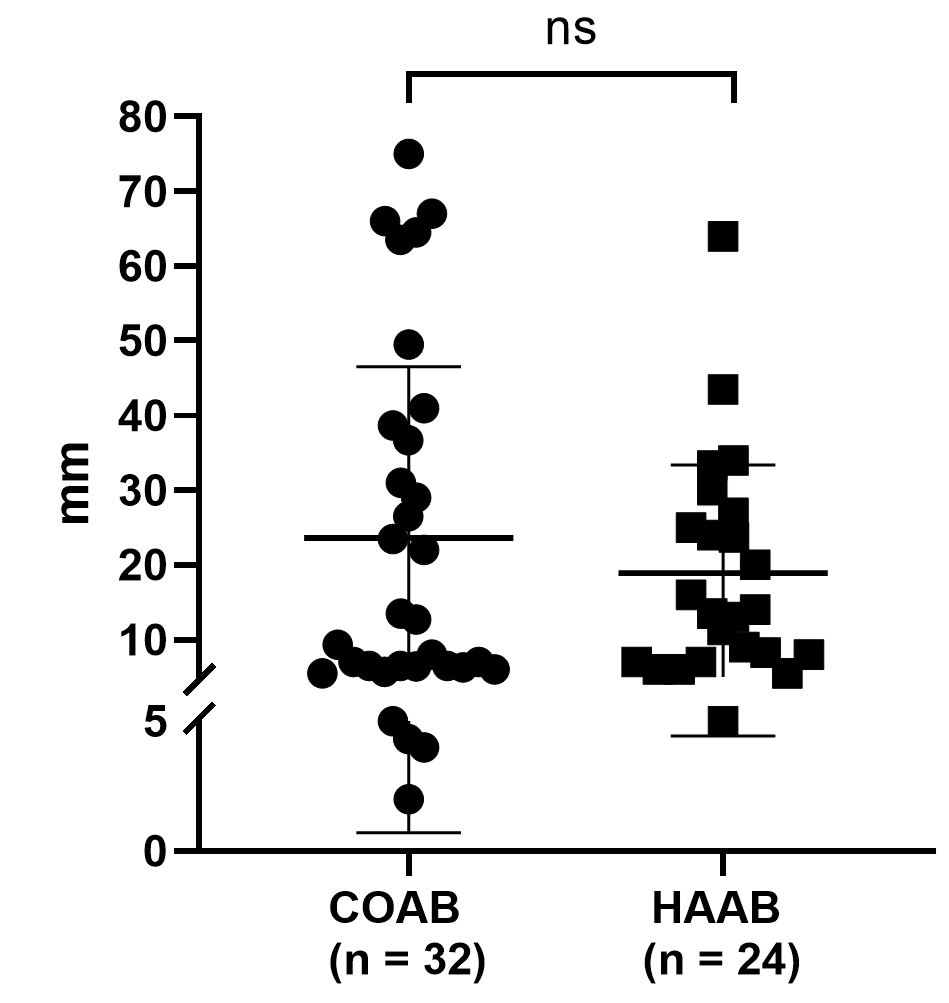
(D) Twitching (E) Swarming**

**FIG. S4 Continued. (D)** Comparison of twitching motility between COAB and HAAB isolates**. (E)** Comparison of swarming motility between COAB and HAAB isolates. *P*-values were calculated using Student's t-test (ns = no significant, p > 0.05).

**(A)**

**
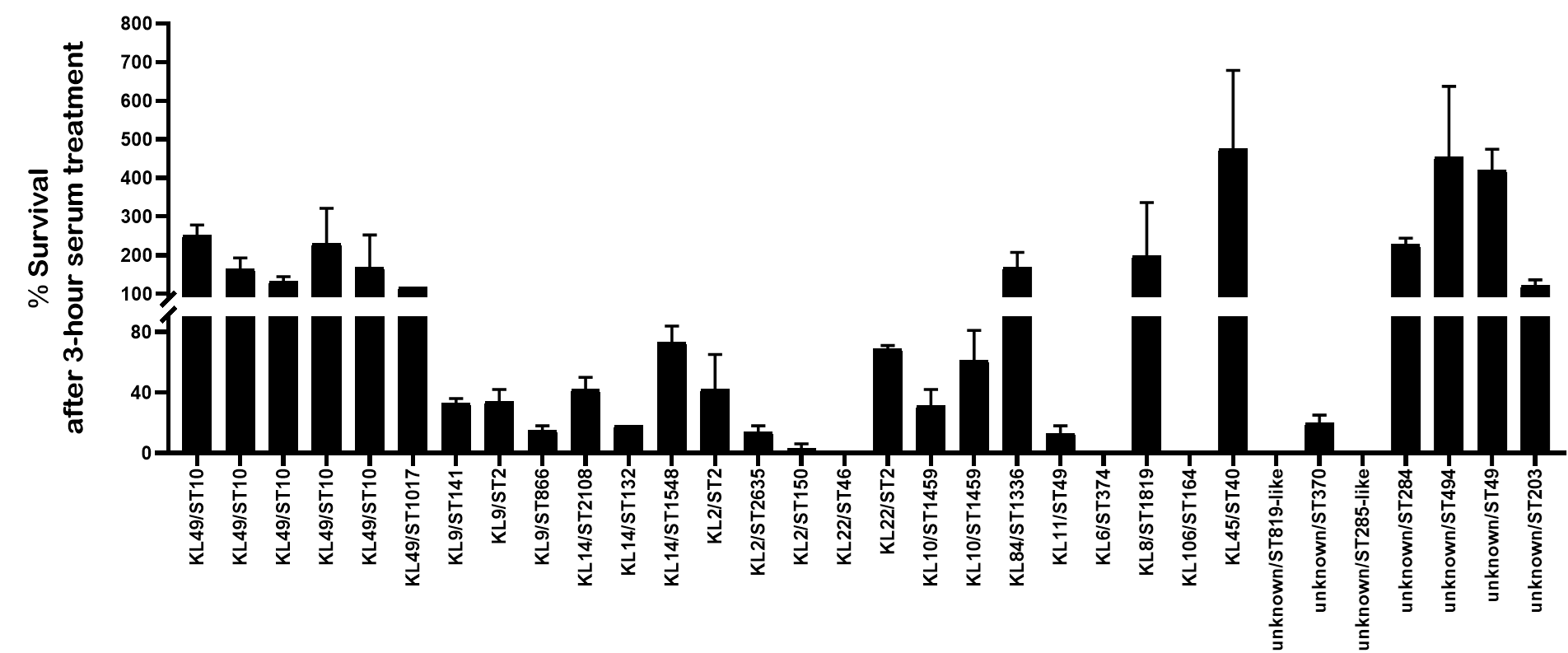
(B)**

**
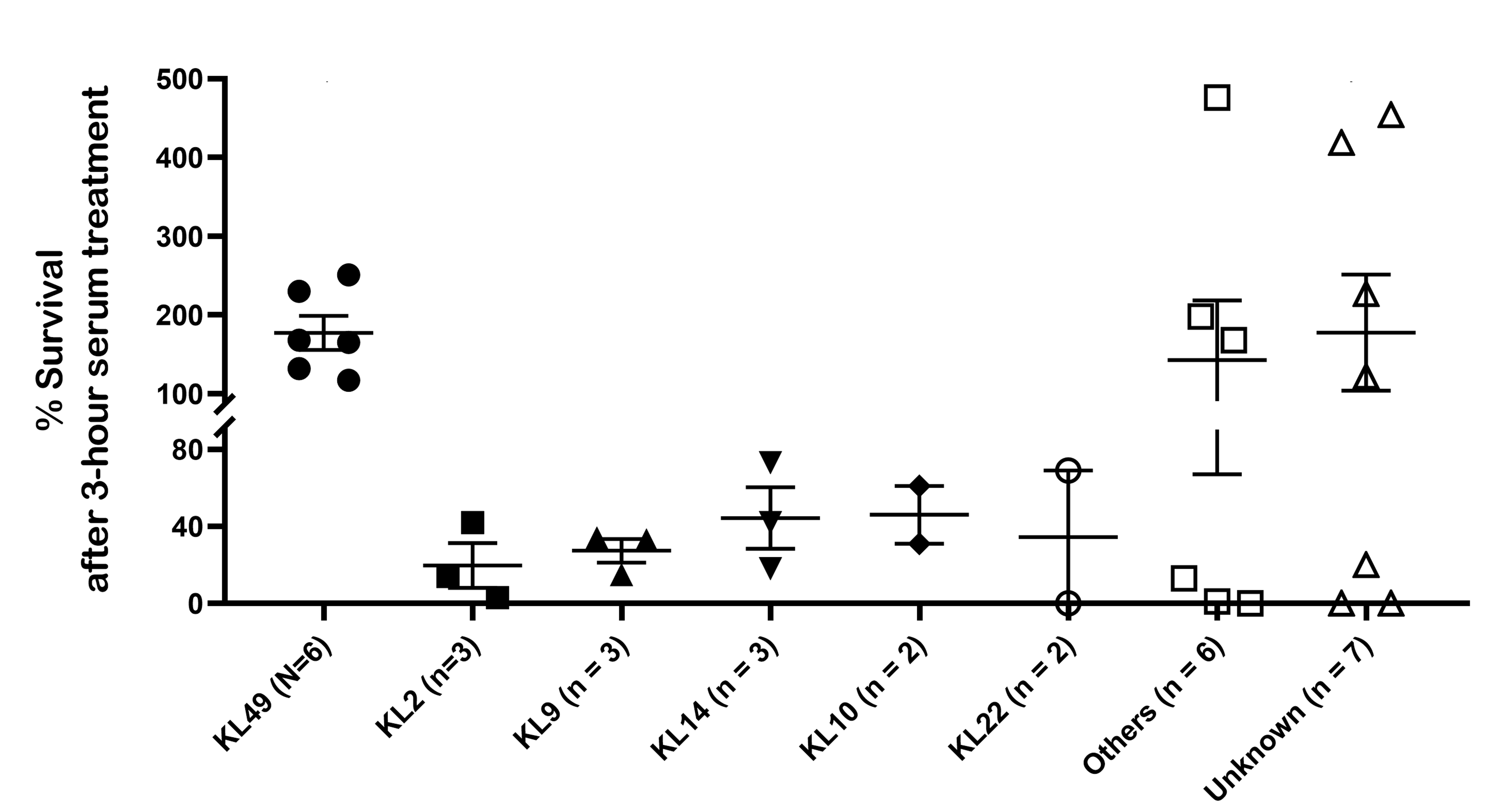
**

**
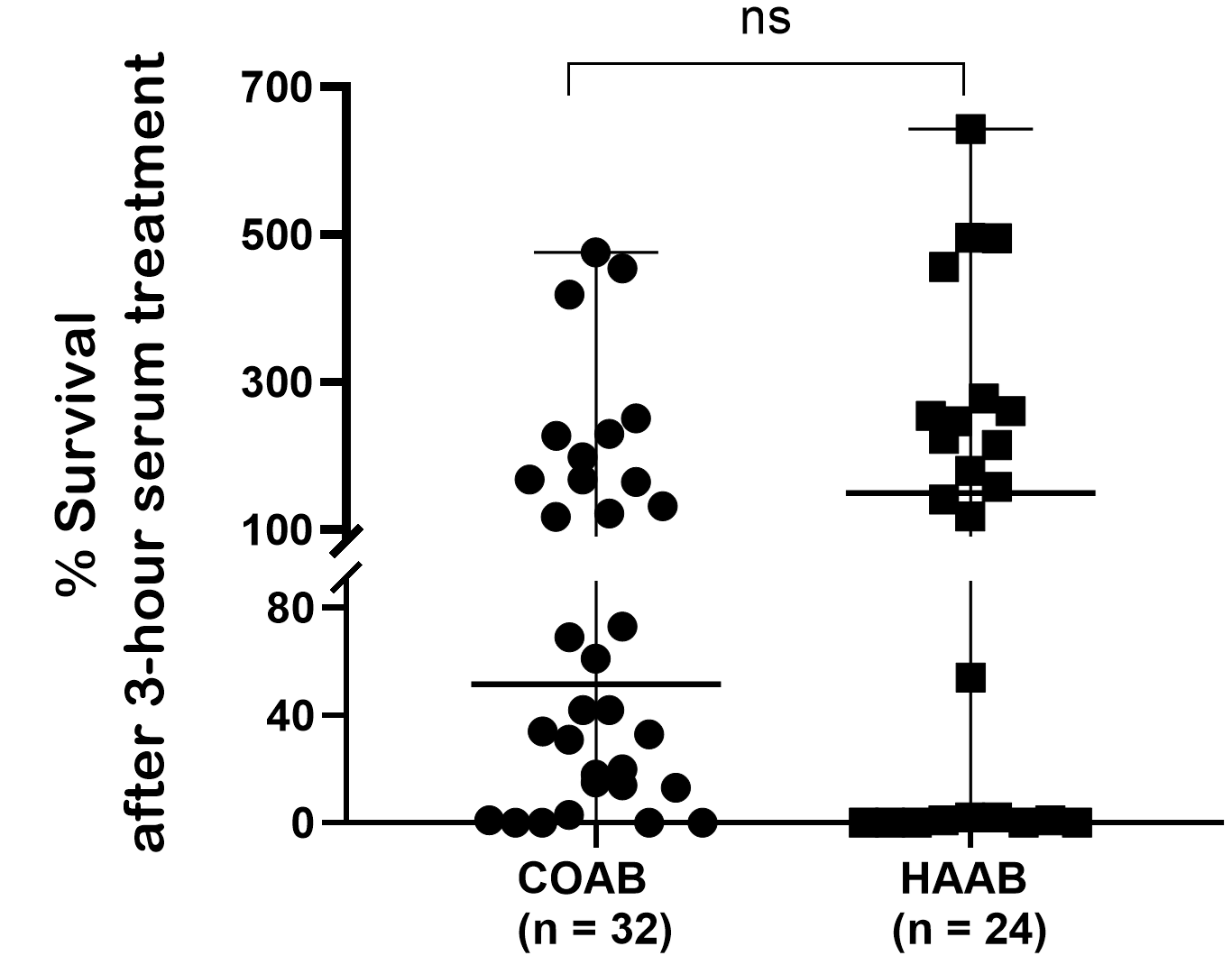
 (D)**

**
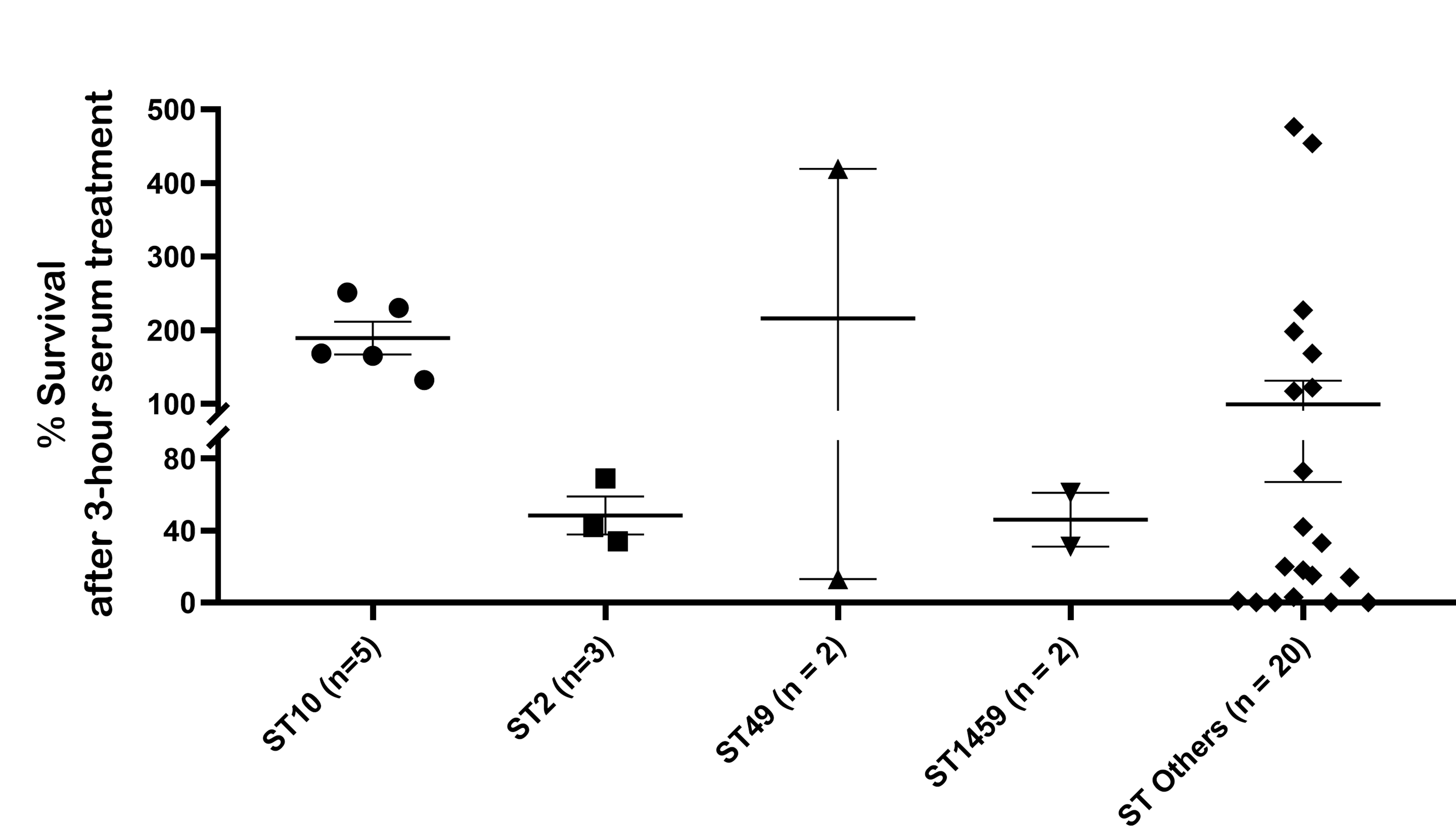
(C)**

**FIG. S5 Serum susceptibility of the COAB isolates. (A)** Percent survival after 3 h of serum treatment**.** Distribution of percent survival after serum treatment for the various **(B)** KL types and **(C)** ST types. **(D)** Comparison of the resistance rate (%) between COAB and HAAB isolates. The *P*-value was calculated using Student's t-test (ns = no significant, *p* > 0.05).


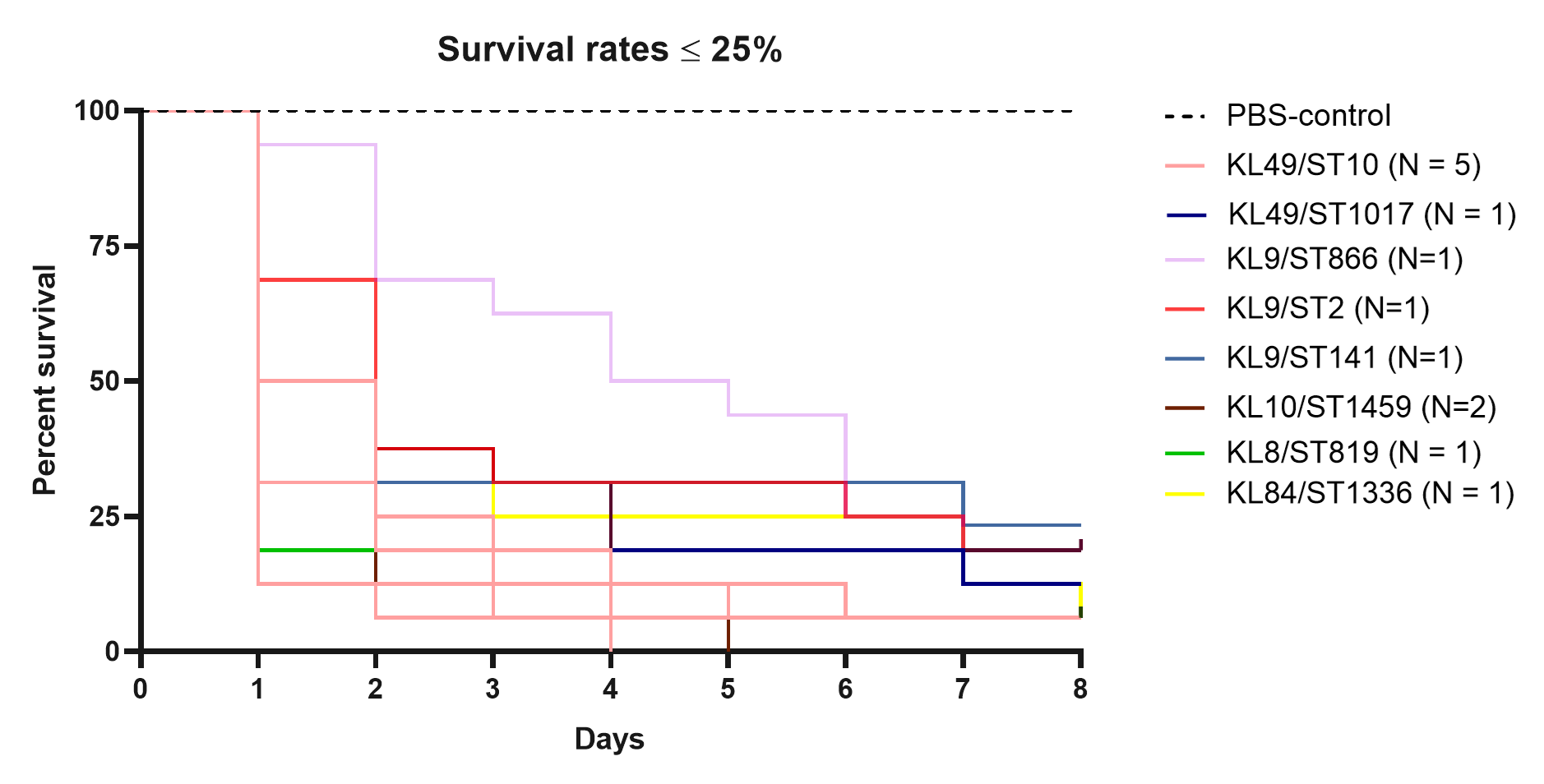
**(A)**


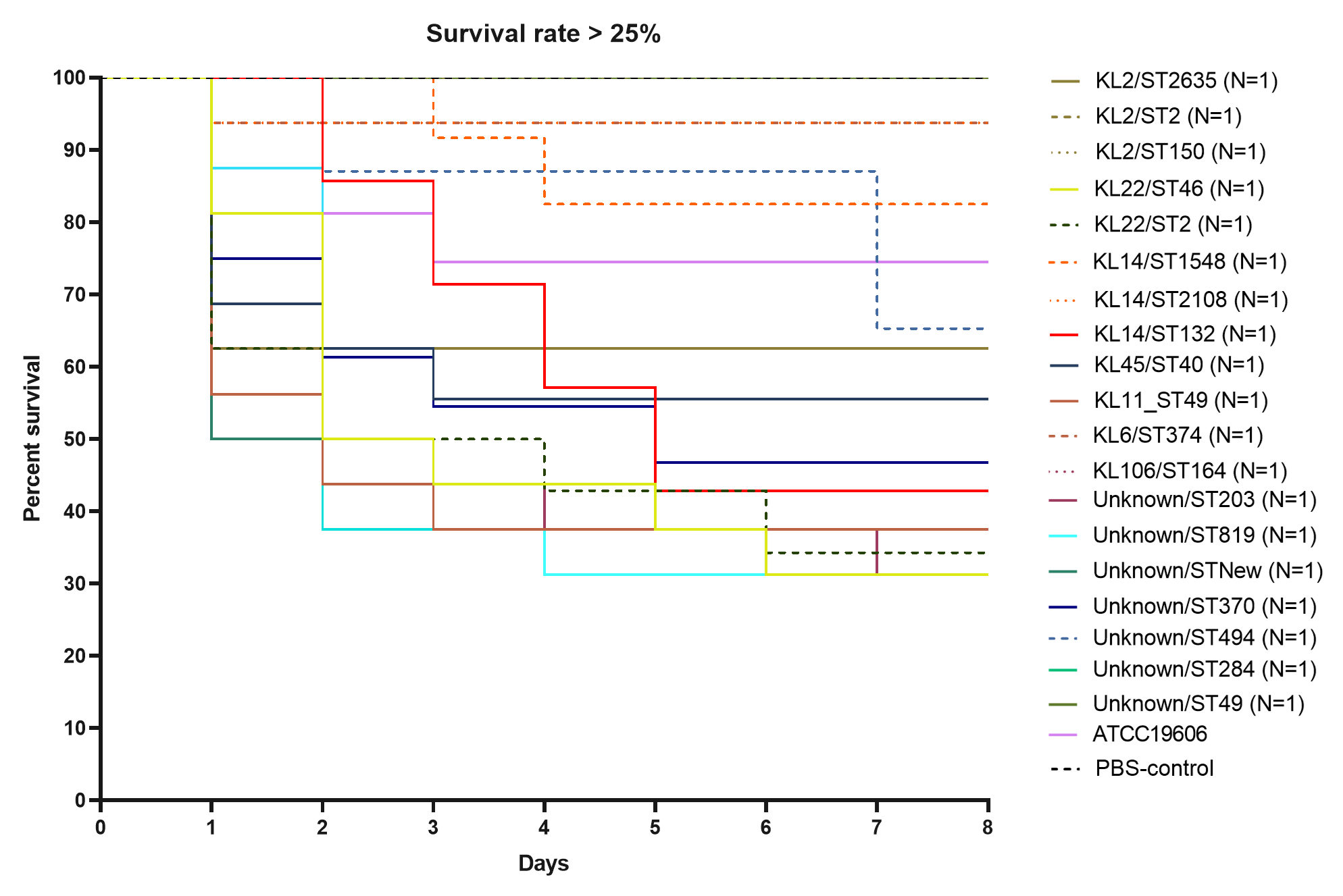


**(B)**


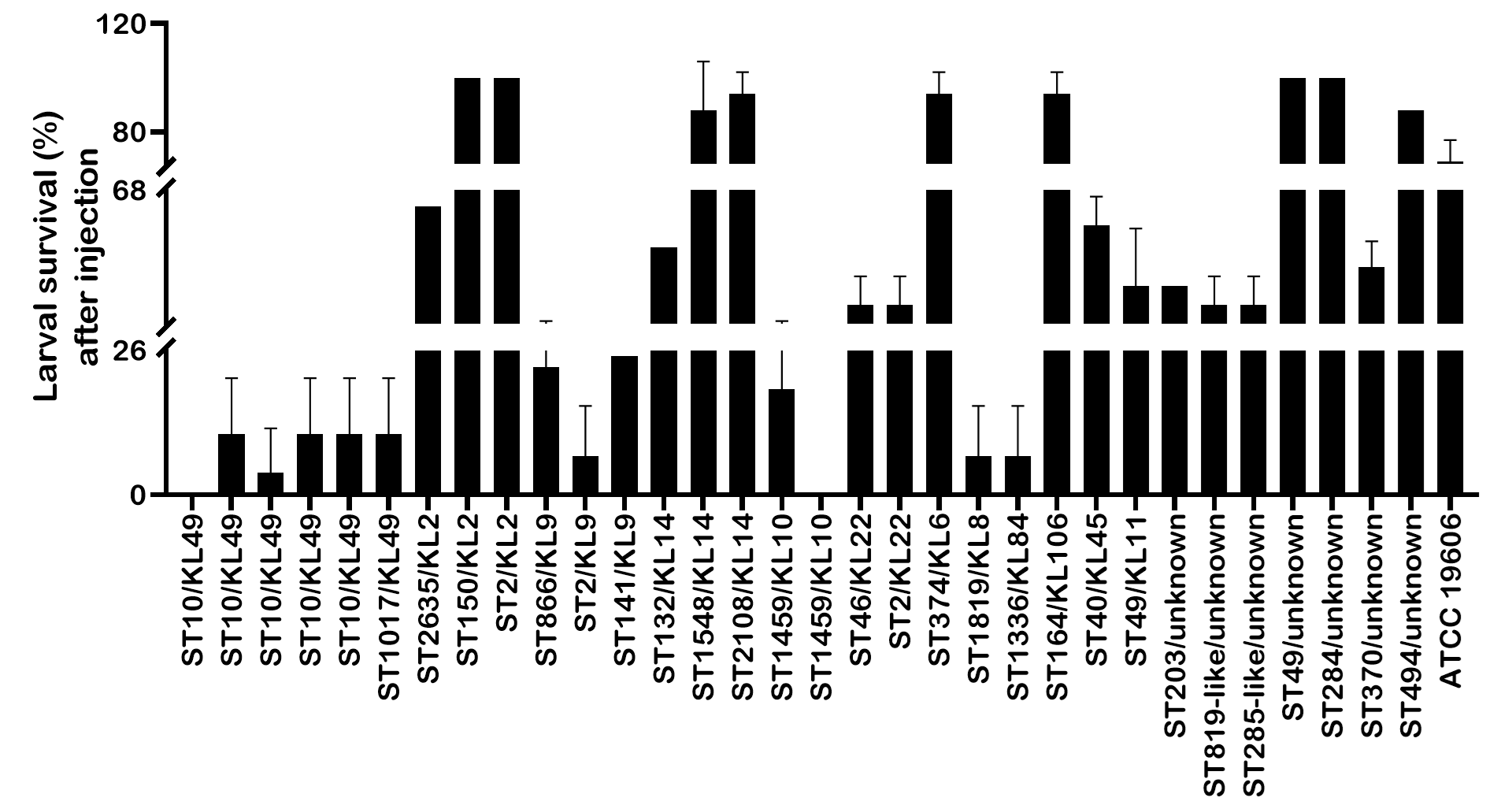


**(C)**

**FIG. S6 *In vivo* virulence in a larval model.** Kaplan–Meier survival curves of *G. mellonella* larvae infected with 32 COAB isolates, resulting in **(A)** ≤25% larval survival and **(B)** in >25% larval survival and ATCC 19606. **(C)** Detailed percent survival of larvae post-injection with the 32 COAB strains and ATCC 19606 reference strain.

**(D)**


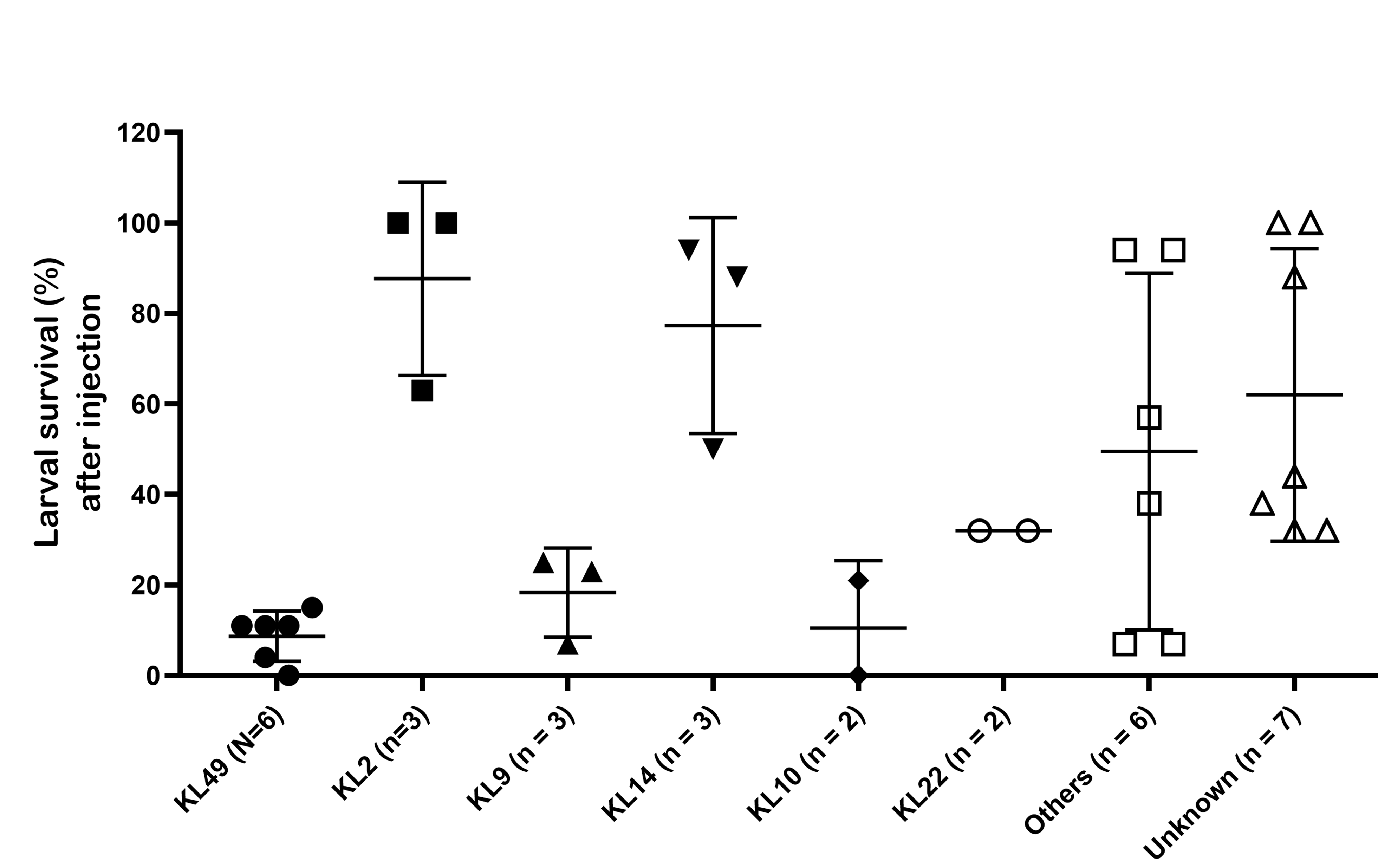


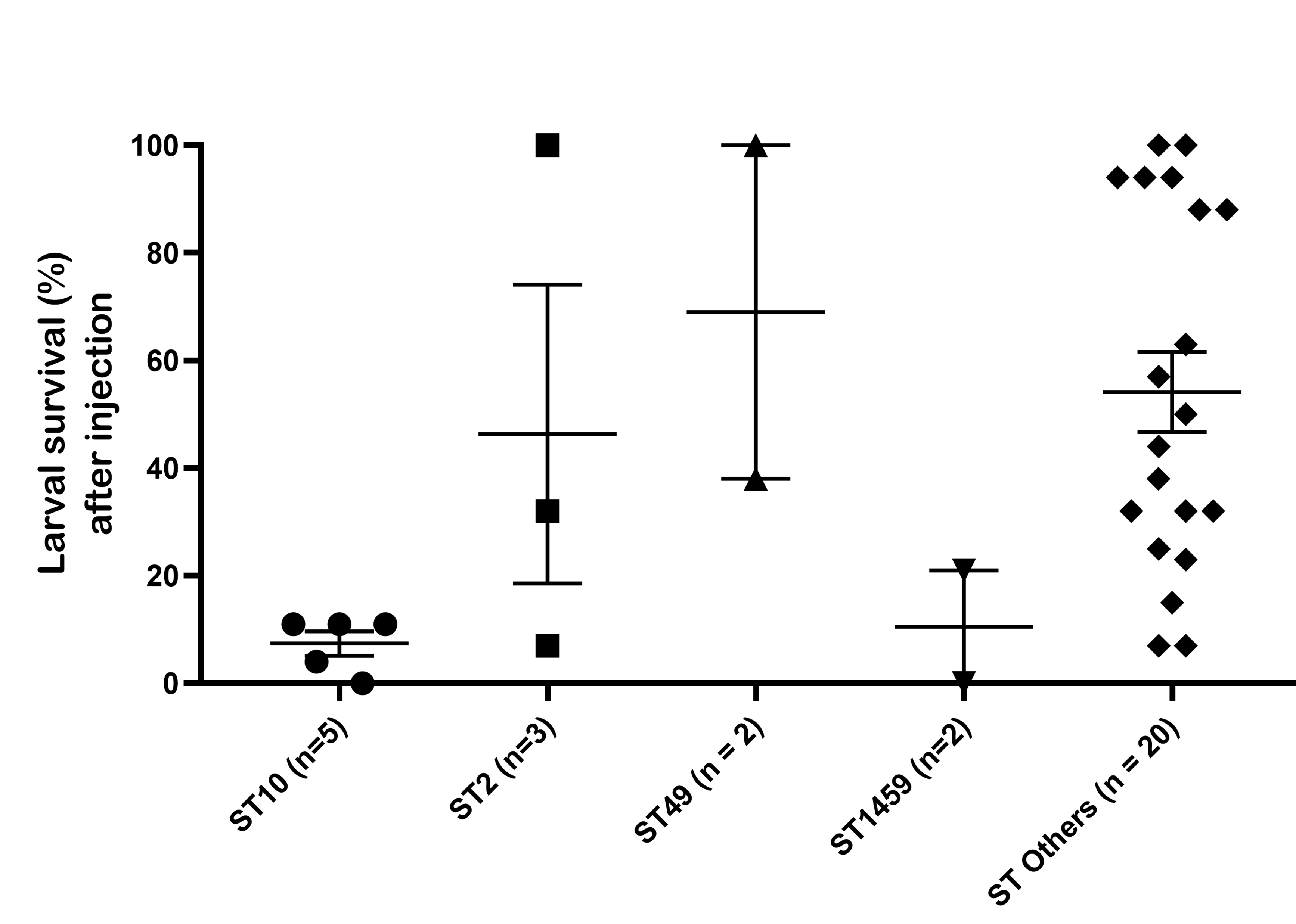
**(E)**


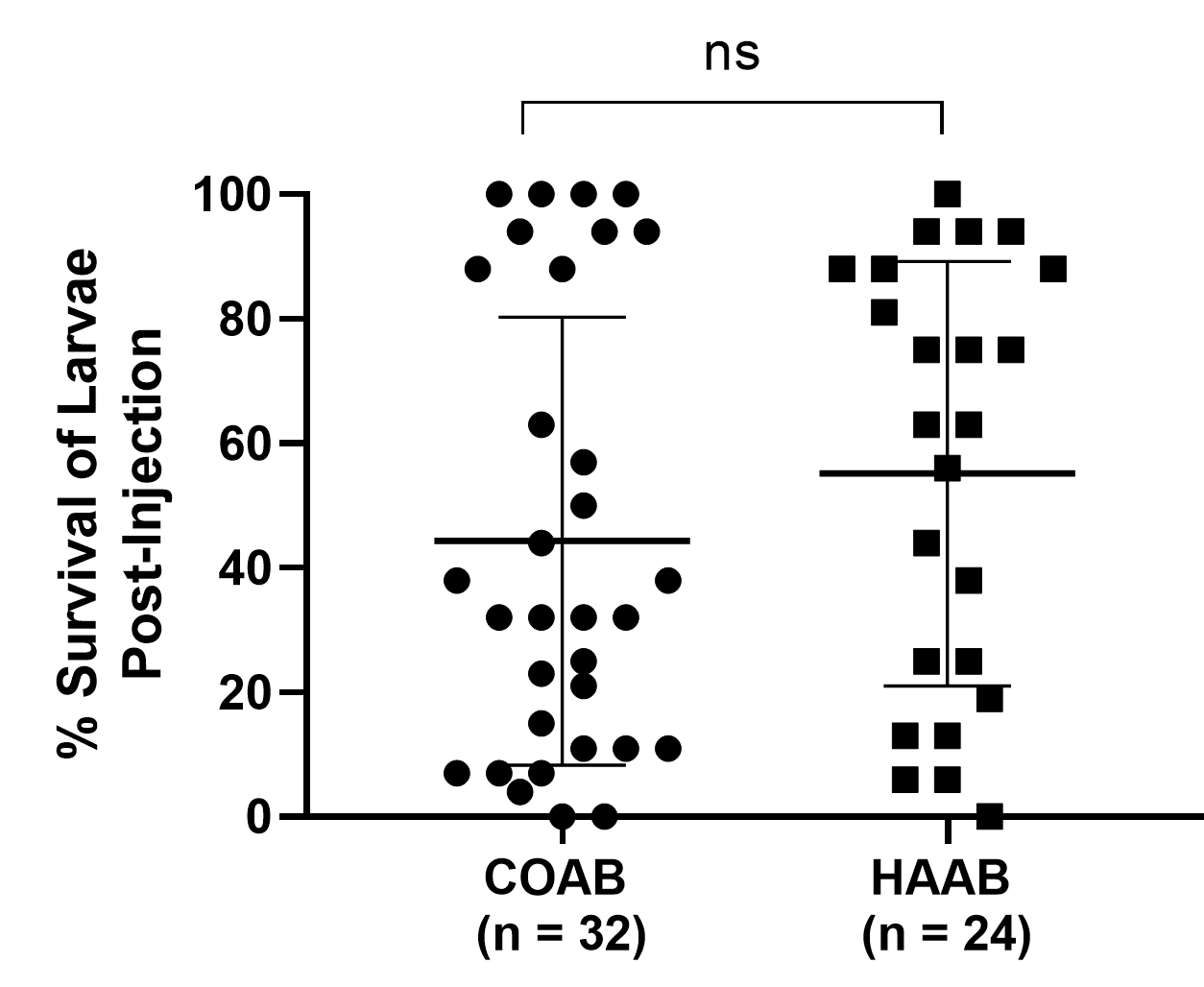
**(F)**

**FIG. S6 *In vivo* virulence in a larval model.** Distribution of percent survival of larvae post-injection for the various **(D)** KL types and **(E)** ST types. **(F)** Comparison of the survival rate (%) between COAB and HAAB isolates. The *P*-value was calculated using Student's t-test (ns = no significant, *p* > 0.05).
