# Supplementary material for: Emerging virulent clones of community-onset Acinetobacter baumannii in Taiwan
Source: Trop Med Health. 2025 Dec 16;53:189. doi: 10.1186/s41182-025-00850-1 (PMC12709753; doi:10.1186/s41182-025-00850-1)
Supplement: Supplementary file 2 — Additional file2 (DOCX 35 KB) [file 41182_2025_850_MOESM2_ESM.docx]

**Table S1.** Allelic profiles based on multi-locus sequence typing (MLST) of 32 COAB isolates using the Pasteur (Pas) and Oxford (Oxf) schemes

| KL type | MLST Pas - 7 allele profiles | | | | | | | ST^Pas^ | MLST Oxf - 7 allele profiles | | | | | | | ST^Oxf^ |
| --- | --- | --- | --- | --- | --- | --- | --- | --- | --- | --- | --- | --- | --- | --- | --- | --- |
|  | *cpn60* | *fusA* | *gltA* | *pyrG* | *recA* | *rplB* | *rpoB* |  | *gltA* | *gyrB* | *gdhB sec* | *recA* | *cpn60* | *gpi* | *rpoD* |  |
| KL49 | 1 | 3 | 2 | 1 | 4 | 4 | 4 | 10 | 1 | 19 | 198 | 12 | 4 | 106 | 2 | New1 |
| KL49 | 1 | 3 | 2 | 1 | 4 | 4 | 4 | 10 | 1 | 15 | 10 | 12 | 4 | 106 | 2 | New2 |
| KL49 | 1 | 3 | 2 | 1 | 4 | 4 | 4 | 10 | 1 | 15 | 34 | 12 | 4 | 106 | 2 | New3 |
| KL49 | 1 | 3 | 2 | 1 | 4 | 4 | 4 | 10 | 1 | 15 | 10 | 12 | 4 | 106 | 2 | New2 |
| KL49 | 1 | 3 | 2 | 1 | 4 | 4 | 4 | 10 | 1 | 15 | 3 | 12 | 4 | 106 | 2 | New4 |
| KL49 | 3 | 3 | 2 | 2 | 9 | 1 | 5 | 1017 | 1 | 15 | 10 | 6 | 1 | 163 | 152 | New5 |
| KL2 | 2 | 2 | 2 | 2 | 2 | 2 | 2 | 2 | 1 | 3 | 3 | 2 | 2 | 97 | 3 | 208 |
| KL2 | 1 | 4 | 2 | 2 | 9 | 1 | 2 | 2635 | 1 | 48 | 127 | 6 | 4 | 371 | 4 | New6 |
| KL2 | 39 | 2 | 2 | 2 | 4 | 27 | 4 | 150 | 1 | 1 | 66 | 12 | 33 | 178 | 41 | 744 |
| KL9 | 26 | 2 | 2 | 1 | 43 | 4 | 5 | 141 | 1 | 15 | 80 | 85 | 28 | 163 | 113 | 2152 |
| KL9 | 2 | 2 | 2 | 2 | 2 | 2 | 2 | 2 | 1 | 3 | 3 | 2 | 2 | 102 | 3 | 218 |
| KL9 | 3 | 2 | 2 | 30 | 3 | 74 | 3 | 866 | 1 | 93 | 42 | 1 | 1 | 284 | 123 | 1436 |
| KL14 | 13 | 1 | 56 | 1 | 7 | 1 | 3 | 2108 | 35 | 12 | 2 | 28 | 22 | 104 | 4 | 2696 |
| KL14 | 3 | 5 | 5 | 1 | 7 | 1 | 4 | 132 | 33 | 31 | 10 | 28 | 1 | 107 | 5 | New7 |
| KL14 | 3 | 2 | 2 | 2 | 3 | 1 | 4 | 1548 | 1 | 34 | 56 | 1 | 1 | 144 | 26 | 1199 |
| KL22 | 5 | 12 | 11 | 2 | 14 | 9 | 14 | 46 | 31 | 33 | 211 | 40 | 16 | 145 | 7 | New8 |
| KL22 | 2 | 2 | 2 | 2 | 2 | 2 | 2 | 2 | 1 | 3 | 3 | 2 | 2 | 99 | 3 | 473 |
| KL10 | 3 | 4 | 2 | 2 | 7 | 2 | 4 | 1459 | 1 | 31 | 2 | 28 | 1 | 99 | 5 | New9 |
| KL10 | 3 | 4 | 2 | 2 | 7 | 2 | 4 | 1459 | 1 | 102 | 80 | 28 | 1 | 166 | 45 | New10 |
| KL6 | 3 | 2 | 2 | 2 | 3 | 4 | 4 | 374 | 1 | 12 | 56 | 1 | 1 | 177 | 26 | 1416 |
| KL8 | 3 | 2 | 2 | 1 | 5 | 1 | 4 | 1819 | 1 | 34 | 26 | 11 | 1 | 163 | 45 | New11 |
| KL11 | 3 | 3 | 6 | 2 | 3 | 1 | 5 | 49 | 2 | 44 | 58 | 1 | 1 | 140 | 41 | New12 |
| KL45 | 1 | 2 | 2 | 2 | 5 | 1 | 14 | 40 | 1 | 56 | 12 | 11 | 4 | 103 | 3 | New13 |
| KL84 | 1 | 3 | 40 | 2 | 7 | 1 | 1 | 1336 | 36 | 31 | 26 | 28 | 4 | 284 | 4 | New14 |
| KL106 | 40 | 3 | 7 | 2 | 40 | 4 | 4 | 164 | 21 | 48 | 58 | 42 | 36 | 166 | 4 | New15 |
| unknown | 3 | 1 | 2 | 2 | 4 | 2 | 14 | 819 | 1 | 17 | 161 | 12 | 1 | 79 | 4 | New16 |
| unknown | 1 | 4 | 3 | 2 | 9 | 1 | 14 | 370 | 18 | 35 | 3 | 6 | 4 | 331 | 50 | New17 |
| unknown | 2 | 52 | 2 | 2 | 9 | 4 | 2 | New | 1 | 3 | 3 | 6 | 2 | 102 | 3 | New18 |
| unknown | 3 | 5 | 2 | 1 | 7 | 1 | 4 | 284 | 1 | 12 | 2 | 28 | 1 | 191 | 4 | New19 |
| unknown | 3 | 3 | 2 | 5 | 4 | 1 | 4 | 494 | 1 | 57 | 204 | 12 | 1 | 103 | 51 | New20 |
| unknown | 3 | 3 | 6 | 2 | 3 | 1 | 5 | 49 | 2 | 44 | 58 | 1 | 1 | 140-like | 41 | New21 |
| unknown | 3 | 4 | 2 | 2 | 7 | 1 | 2 | 203 | 1 | 34 | 80 | 28 | 1 | 163 | 4 | New22 |

**Table S2. Shared and unique Pasteur sequence types (STs) in community and hospital isolates**

|  | Community-onset  *A. baumannii* (COAB) | Hospital-acquired *A. baumannii*  (HAAB) |
| --- | --- | --- |
| STs Unique | ST10, ST40, ST46, ST49, ST150, ST164, ST203, ST284, ST370, ST494, ST819, ST866, ST1017, ST1336, ST1459, ST1548, ST1819, ST2108, ST2365 | ST129, ST130, ST131, ST133, ST134, ST135, ST136, ST137, ST138, ST139, ST140, ST142, ST143, ST195, ST367, ST375, ST376, ST377, ST378, ST379, ST380, ST381, ST382, ST383, ST384, ST386, ST387, ST388, ST390, ST391, ST392, ST393, ST394, ST395, ST396, ST397, ST398, ST399, ST719, ST720, ST721, ST722, ST723, ST724, ST725, ST726, ST727, ST728, ST2462 |
| STs Shared by COAB and HAAB | ST2, ST132, ST141, ST374 | |

Note: Only sequence types (STs) determined using the Pasteur MLST scheme are shown. The comparison of unique and shared STs is based on data from Fig. S1A and S1B. STs detected exclusively in either CAAB or HAAB groups are listed as unique. Shared STs were found in both groups.

**Table S3.** Comparison of the antimicrobial susceptibilities of COAB and HAAB isolates

| **Classes of antibiotics** | **Antimicrobial agents** | **^a^Non-susceptible, number (%)** | | ***p–*value** |
| --- | --- | --- | --- | --- |
|  |  | **COAB (n=32)** | **HAAB (n=24)** |  |
| Aminoglycosides | Amikacin | 3(9.38) | 19(79.17) | **<0.0001** |
|  | Gentamicin | 4(12.5) | 22(91.67) | **<0.0001** |
| β-lactams | Cefepime | 4(12.5) | 22(91.67) | **<0.0001** |
|  | Ceftazidime | 4(12.5) | 23(95.83) | **<0.0001** |
| β-lactams /  β-lactamase inhibitor | Piperacillin-tazobactam | 5(15.63) | 22(91.67) | **<0.0001** |
|  | Ampicillin-sulbactam | 2(6.45)^b^ | 19(79.17) | **<0.0001** |
| Fluoroquinolones | Ciprofloxacin | 4(12.5) | 22(91.67) | **<0.0001** |
| Third-generation cephalosporin /  β-lactamase inhibitor | Cefoperazone-sulbactam | 0(0)^c^ | 18(81.82)^c^ | **<0.0001** |
| Carbapenems | Imipenem^#^ | 1(3.13) | 19(79.17) | **<0.0001** |
|  | Meropenem^#^ | 1(3.13) | 18(75) | **<0.0001** |
| Tetracyclines | Tigecycline | 1(3.13) | 9(37.5) | **0.0012** |
| Polymyxins | Colistin^#^ | 0(0) | 1(4.17) | 0.4286 |
| **Carbapenem resistance** | | 1(3.13) | 19(79.17) | **<0.0001** |
| **Multidrug resistance** | | 1(3.13) | 16(66.67) | **<0.0001** |

a: Included intermediate and resistant strains

b: Data on one isolate were not available.

c: Data on two isolates were not available.

^#^Minimum inhibitor concentration was determined using the broth microdilution method according to Clinical and Laboratory Standards Institute CLSI [31]. Fisher’s Exact Test was used to assess differences between two groups. Statistically significant results (p < 0.05) are indicated in bold.

**Table S4.** Power calculation using G*Power 3.1.9.7

|  | Power | | |
| --- | --- | --- | --- |
| Effect size d | t test  (n1=6, n2=26)^a^ | t test  (n1=5, n2=27)^b^ | Chi-square test (n=32) |
| 0.1 | 0.055 | 0.054 | 0.087 |
| 0.3 | 0.096 | 0.090 | 0.396 |
| 0.5 | 0.181 | 0.163 | 0.807 |
| 0.7 | 0.309 | 0.274 | 0.977 |
| 0.9 | 0.467 | 0.415 | 0.999 |

Note: a, KL49(n=6) and non-KL49 (n=26); b, ST10 (n=5) and non-ST10 (n=27); G*Power value below 0.80 generally indicates that a statistical analysis is likely underpowered, suggesting that the sample size may be too small or the expected effect size too modest to reliably detect significant results.
